# Early diagnosis of autism across developmental stages through scalable and interpretable ensemble model

**DOI:** 10.3389/frai.2025.1507922

**Published:** 2025-05-30

**Authors:** Nasirul Mumenin, Maisha Mumtaz Rahman, Mohammad Abu Yousuf, Farzan M. Noori, Md Zia Uddin

**Affiliations:** ^1^Department of Computer Science and Engineering, Bangladesh University of Business and Technology, Dhaka, Bangladesh; ^2^Department of Electrical and Electronics Engineering, Islamic University of Technology, Dhaka, Bangladesh; ^3^Institute of Information Technology, Jahangirnagar University, Dhaka, Bangladesh; ^4^Department of Informatics, University of Oslo, Oslo, Norway; ^5^Department of Sustainable Communication Technologies, SINTEF (Norwegian: Stiftelsen for Industriell og Teknisk Forskning) Digital, Oslo, Norway

**Keywords:** Autism Spectrum Disorder, ensemble model, uncertainty analysis, explainable AI, Monte Carlo Dropout, SHAP

## Abstract

Autism Spectrum Disorder (ASD) is a multifaceted neurodevelopmental condition that challenges early diagnosis due to its diverse manifestations across different developmental stages. Timely and accurate detection is essential to enable interventions that significantly enhance developmental outcomes. This study introduces a robust and interpretable machine learning framework to diagnose ASD using questionnaire data. The proposed framework leverages a stacked ensemble model, combining Random Forest (RF), Extra Tree (ET), and CatBoost (CB) as base classifiers, with an Artificial Neural Network (ANN) serving as the meta-classifier. The methodology addresses class imbalance using Safe-Level SMOTE, dimensionality reduction via Principal Component Analysis (PCA), and feature selection using Mutual Information and Pearson correlation. Evaluation on publicly available datasets representing toddlers, children, adolescents, adults, and a merged dataset (Combining children, adolescents, and adults dataset) demonstrates high diagnostic accuracy, achieving 99.86%, 99.68%, 98.17%, 99.89%, and 96.96%, respectively. Comparative analysis with standard machine learning models underscores the superior performance of the proposed framework. SHapley Additive exPlanations (SHAP) were used to interpret feature importance, while Monte Carlo Dropout (MCD) quantified uncertainty in predictions. This framework provides a scalable, interpretable, and reliable solution for ASD screening across diverse populations and developmental stages.

## 1 Introduction

Autism Spectrum Disorder (ASD) is a complex neurodevelopmental condition characterized by a wide range of symptoms and severity levels, affecting communication, behavior, and social interactions. The early and accurate diagnosis of ASD is crucial for initiating appropriate interventions that can significantly improve the quality of life for individuals with ASD and their families. However, the heterogeneous nature of ASD, combined with overlapping symptoms with other developmental disorders, poses substantial challenges to its diagnosis.

Several biomarkers have received attention because of their ability to detect individuals who are at increased risk of developing ASD. Questionnaire-based screening tools are among the most widely used methods for preliminary ASD assessment. These tools generate rich datasets that encode valuable information on individuals' behavioral and developmental characteristics. However, such data's complexity and high dimensionality necessitate sophisticated analytical techniques to extract meaningful patterns and insights. Comprehensive diagnostic assessments for ASD, such as ADOS (Bastiaansen et al., 2011) or ADI-R (De Bildt et al., 2004), can be time-consuming, often requiring multiple sessions over several days. Diagnoses are subject to interpretation by the clinician, which leads to potential variability between evaluators. This subjectivity can affect the consistency and reliability of the diagnosis. The need for specialized training to conduct assessments limits the availability of qualified professionals, especially in underserved or rural areas.

Recent years have witnessed an increase in the number of establishments exploring the use of ML techniques to aid in the early detection of ASD, with the aim of complementing traditional diagnostic processes with objective data-driven approaches (Hasan et al., 2022; Mumenin et al., 2023, 2024). ML algorithms can analyze complex and high-dimensional data from various sources, including genetic, neuroimaging, and behavioral data. Questionnaire-based tools, in particular, provide a valuable resource for ML models. Research in areas such as medical diagnosis, optimization, and pattern recognition has demonstrated the efficacy of these hybrid approaches (Talukder et al., 2023; Mumenin et al., 2025; Choudhury et al., 2025). While traditional ML methods have demonstrated success in ASD detection, they often fall short in three critical areas: (1) handling class imbalance effectively, (2) ensuring interpretability of predictions, and (3) providing reliable uncertainty estimates to support clinical decision-making. Furthermore, most existing methods focus on a single algorithmic approach, limiting their ability to fully exploit the diversity of complex data patterns inherent in questionnaire-based ASD assessments.

This paper introduces an innovative methodology that combines stacked ensemble model (EM), XAI, and Uncertainty Analysis (UA) to improve the precision and dependability of ASD classification. The EM that has been proposed not only enhances the accuracy of classification but also integrates mechanisms for evaluating and managing uncertainty. This facet is frequently disregarded in conventional models. The proposed model capitalizes on the advantages of multiple base classifiers, each providing distinct viewpoints in identifying ASD, thus generating a comprehensive, multidimensional feature space. Using a metaclassifier to integrate these base classifiers, the ultimate prediction is guaranteed to represent an exhaustive examination of the underlying patterns present in the data.

In addition, the proposed model places significant importance on interpretability, a critical aspect in medical applications where understanding the reasoning behind classifications is crucial to establishing trust and facilitating subsequent analysis. An essential advance of this research is to integrate uncertainty consciousness into the framework. Acknowledging that a specific sample might exhibit equivocal or deceptive characteristics, our model incorporates a confidence metric into its predictions to offer significant insights into its decision-making methodology. This functionality is critical for end-users and allows the model to be continuously enhanced by flagging instances with high uncertainty for additional investigation or manual review.

The main contributions of this study are:

- Development of a stacked EM model that effectively classifies ASD across multiple age groups. Safe-Level SMOTE ensures balanced data representation, improving model generalization and mitigating class imbalance issues.
- Incorporation of SHAP for model interpretability, enabling the identification of key features influencing predictions. SHAP plots provide insights into the factors responsible for particular classifications, fostering trust and understanding of the model's decisions.
- Utilization of MCD for uncertainty estimation, allowing the model to quantify its confidence in predictions. This enhances the reliability of the framework, addressing a critical need for dependable tools in clinical decision-making.
- The model is tested and validated on multiple publicly available datasets representing diverse developmental stages (toddler, child, adolescent, and adult) and an integrated dataset. Evaluation through standard metrics (accuracy, precision, recall, and F1-score) demonstrates the robustness and scalability of the proposed approach.

The rest of the paper is organized as follows: Section 2 presents a detailed literature review. Section 3 discusses the architecture and methodology of the study, along with the tools and techniques that were implemented. In Section 4, we analyze the experimental results and provide a performance comparison of the proposed method. Section 5 describes the implementation and findings of explainable artificial intelligence (XAI). Section 6 explains the use of Monte Carlo Dropout (MCD) and how it enhances the results. Finally, Section 7 concludes the paper with a concise discussion of the drawbacks and potential future directions of the work.

## 2 Literature review

A significant number of researchers have utilized ML-based models to diagnose ASD. Hasan et al. (2022) detected ASD in individuals of various age groups. The authors demonstrated that ML-based predictive models are effective instruments for this endeavor. Mukherjee et al. (2023) presented three frameworks with ML models to detect ASD among children, toddlers, and adults. They explored the facial image-based and questionnaire-based techniques for the detection of ASD. Bala et al. (2022) introduced an ML model that analyzes ASD data across various age groups and accurately identifies ASD. For such purpose, datasets were collected on ASD from toddlers, children, adolescents, and adults. Afterwards, various classifiers were implemented on these datasets, and evaluation metrics were used to assess their efficacy. Kamma et al. (2022) proposed a Light Gradient Boost (LGB) based model to classify ASD and a Random Search for hyperparameter optimization. A synthesis of three publicly accessible datasets comprising records of ASD in infants, adolescents, and adults was used. Devika Varshini and Chinnaiyan (2020) evaluated the efficacy of a range of ML algorithms and preprocessing methods in the classification of medical datasets intending to forecast early autism symptoms in both toddlers and adults.

Stirling et al. (2021) examined the application of a Self-Organizing Fuzzy classifier and the UCI “Autism Screening Adult” dataset to predict whether an individual is more likely to have ASD and therefore merits a higher priority for subsequent testing and diagnosis. Using an efficient ensemble classification method, Haroon and Padma (2022) sought to detect and diagnose Parkinson's Disease and ASD in their early stages. A delayed or erroneous diagnosis could endanger the life of the patient. Consequently, early and accurate detection has been the primary objective of this research. In their study, Hasan et al. (2021) collected ASD data from both toddlers and adults, implemented seven distinct classification techniques, and evaluated the results. Using statistical and ML techniques, they computed the significant and associative features of both datasets. Additionally, they have identified characteristics that may be utilized to classify children with ASD. The ML architecture proposed by Uddin et al. (2023) was implemented to produce more accurate and efficient outcomes in the rapid diagnosis of ASD. FT techniques were implemented on the ASD samples and the modified dataset was evaluated to determine the effectiveness of numerous classifiers. The significant characteristics of normal and ASD individuals in Bangladesh were investigated (Satu et al., 2019). Individual samples were obtained from parents of children aged 16 to 30 months from various residents through the utilization of Autism Barta applications, both in the field and via the Internet. An evaluation was conducted on various tree-based techniques in order to determine their optimal classifier. Akter et al. (2021b) introduced an improved ML model that exhibits enhanced accuracy in autism detection. An examination was conducted on the correlation between individual and highly co-linear features in these datasets. To assess the symptoms of ASD, Thabtah et al. (2018) devised a rules-based ML (RML) methodology. They discovered that RML enhances the efficacy of the classifier. Abbas et al. (2018) combined ADI-R and ADOS ML methodologies in a unified assessment and resolved the scarcity, sparsity, and data imbalance challenges by implementing feature encoding techniques. In addition, an alternative investigation conducted by Thabtah et al. (2018) and Thabtah (2019) introduced Variable Analysis (VA), a computational intelligence (CI) method that used LR, decision trees (DT), and SVM to generate accurate prognoses and diagnoses for ASD. The VA method illustrated correlations between features and between features.

Researchers have also used various Deep Learning (DL) techniques to diagnose ASD. Mujeeb Rahman and Monica Subashini (2022) examined the accuracy with which DNN-based models identified autism in toddlers using the QCHAT datasets that had previously been collected. Two distinct DL models were developed to process the two iterations of the QCHAT and QCHAT-10 datasets. Mohanty et al. (2021) made an effort to integrate Principal Component Analysis (PCA) to reduce feature dimensions, after which DNN was utilized to classify the type of ASD. The results of the experiment suggest that the combination of PCA and DNN yields clinically acceptable results in accurately identifying ASD. Garg et al. (2022) introduced a hybrid methodology that combines XAI and DL to identify the most influential features for the timely and accurate prognosis of ASD. The suggested framework provides enhanced predictive capabilities and clinical recommendations for predicted outcomes, serving as a crucial tool for the early and improved identification of ASD traits in toddlers. Hajjej et al. (2024) proposed a two-stage framework: In the initial stage, a collection of ML models, such as a random forest ensemble and XGBoost classifiers, are utilized to accurately identify Autism Spectrum Disorders. Identifying appropriate teaching methods for children with ASD through an evaluation of their verbal, physical, and behavioral performance is the focus of the second phase of the research. Utilizing an EL approach, Kampa et al. (2022) developed a model for diagnosing ASD in datasets about children and toddlers. This method serves as a supplement to the traditional single-learning approaches. They achieved favorable performance outcomes by employing feature selection and an EM.

Among the numerous ML and DL methodologies, EM has demonstrated the most potential (Ganaie et al., 2022; Rincy and Gupta, 2020). Ensemble learning (EL) is a technique that combines multiple models to enhance the precision, resilience, and applicability of predictions. Despite progress in detection and classification methodologies, persistent challenges remain, specifically in managing the vast quantity and diverse range of newly discovered ASD samples. An increasing demand exists for models that possess the critical qualities of high accuracy, interpretability, and uncertainty tolerance. DL models, specifically those built upon ANNs, are frequently called “black boxes” due to their complex architectures and the opaque manner in which they produce results (Samek et al., 2017). XAI aims to address this disparity by offering stakeholders a deeper understanding of how these intricate models operate, thus cultivating confidence and empowering them to understand, rely on and efficiently administer AI solutions. Moreover, UA plays a critical role in DL as it enables the evaluation of the dependability and resilience of model predictions (Abdar et al., 2021). DL models may occasionally generate overly optimistic forecasts due to spurious correlations or an inadequate understanding of the data, which can result in decisions that are potentially risky and overly confident (Gawlikowski et al., 2023). Practitioners can enhance the prudence and knowledge of decision-making by identifying instances in which the model's output may be unreliable through the analysis and quantification of prediction uncertainty.

## 3 Proposed methodology

**Figure 1:**
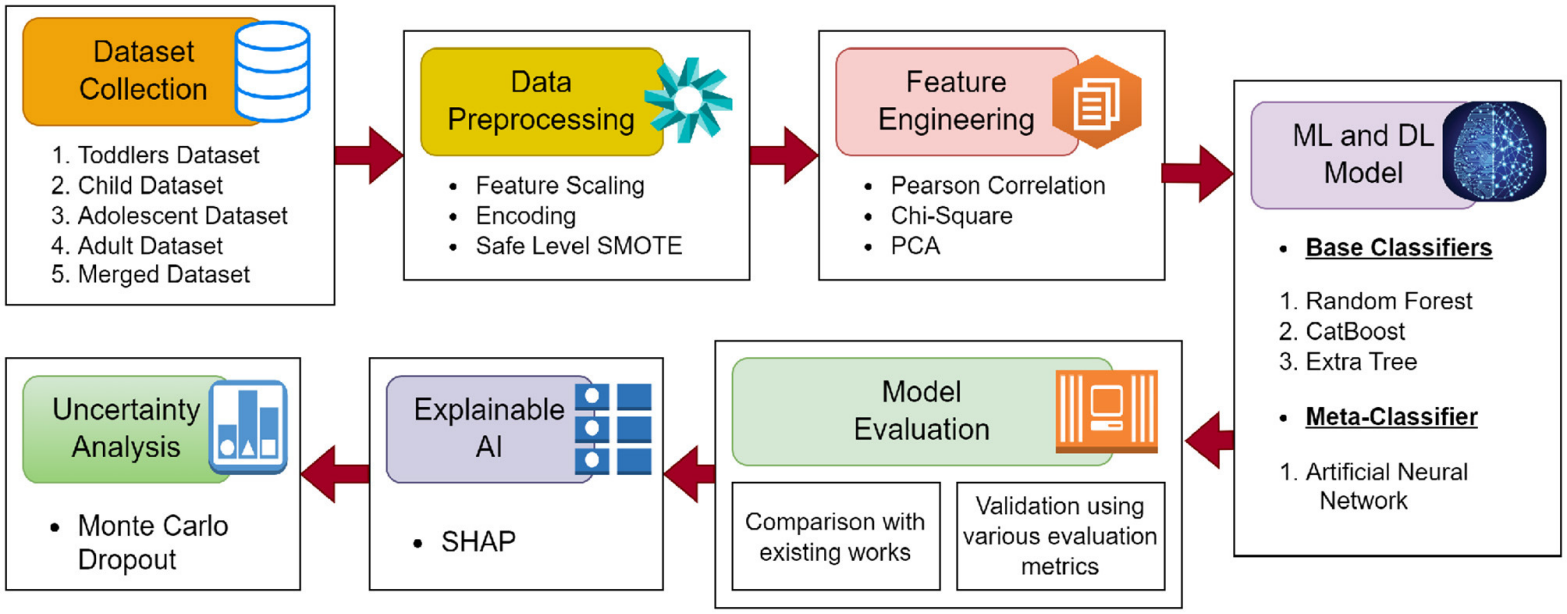
The workflow of the proposed methodology.

The workflow of proposed methodology is shown in Figure 1.

### 3.1 Dataset

The four ASD datasets (Toddlers, Adolescents, Children, and Adults) were obtained from repositories that are accessible to the public: UCI ML and Kaggle (Thabtah, 2018, 2017a,b; Tabtah, 2017). The ASDTests smartphone application, which employs the QCHAT-10 and AQ-10 for ASD screening in toddlers, children, adolescents, and adults, was developed by Thabtah et al. (2018) and Thabtah (2019). An affirmative diagnosis of ASD is indicated by an ultimate score of 6 out of 10 on a scale of zero to ten, which is calculated for each individual by application. Furthermore, the ASDTests application provides access to ASD data, and open-source databases are being created to aid in the investigation of this field. In conclusion, three datasets (child, adolescent, adult) have been merged to form a single dataset. The Adolescents, Children, and Adults datasets were merged primarily because they share identical feature sets, facilitating seamless integration into a unified dataset. Our primary motivation for merging these datasets was to build a robust model capable of generalizing across a broader developmental spectrum, rather than developing multiple separate age-specific models. While explicit statistical analyses (e.g., distributional comparisons) were not performed, the identical nature of the features and consistent data-collection procedures across these datasets and the experimental results proved that merging did not adversely impact the model's performance. The data sets used in this study have been classified as follows: toddler, child, adolescent, adult, and merged.

### 3.2 Data preprocessing

Data preparation is a critical phase in the ML pipeline, as it involves cleaning, transforming, and normalizing data to make it suitable for analysis and training models. Missing values have been found in “child (4)”, and “adult (2)” the dataset's “age” columns. The missing values were replaced using the median imputation technique. Since most ML models are based on mathematical equations, categorical data must be converted to numerical data to avoid complications. So, we encoded the values in the column “ASD traits” that contained categorical data (No, Yes) into numerical values (0, 1).

**Figure 2:**
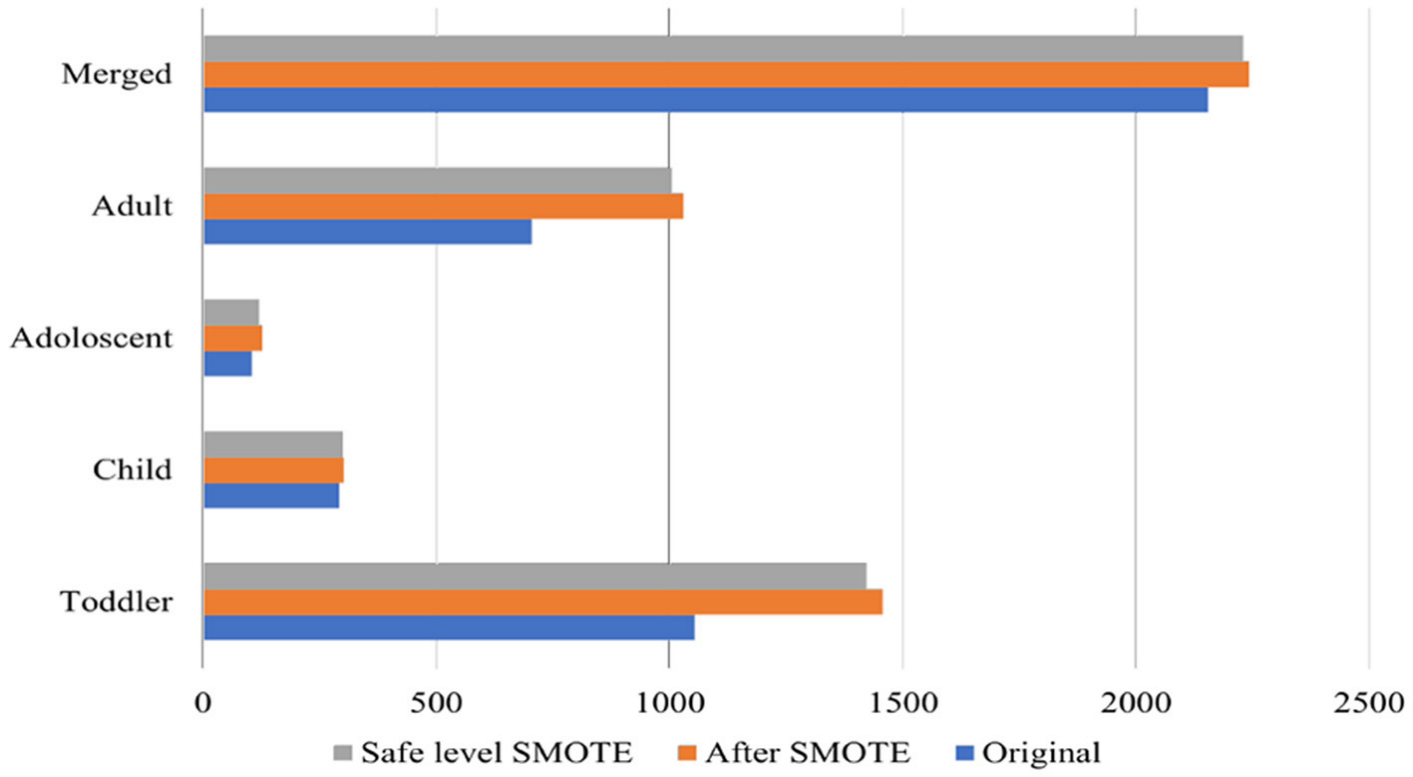
The number of data in original datasets, after applying SMOTE, and safe-level-SMOTE.

To address the class imbalance, Safe-level-SMOTE was utilized. The Synthetic Minority Oversampling Technique (SMOTE) is a method used to address class imbalance in datasets, particularly in the context of supervised learning (Chawla et al., 2002). Figure 2 shows the size of the original datasets, and the size after applying SMOTE, and Safe-level-SMOTE. It works by creating synthetic samples from the minority class rather than copies, which helps overcome the overfitting problem of random oversampling. When constructing predictive models, it is critical to comprehend the significance of each feature in relation to the target variable. A productive approach to assess this level of importance is the computation of Mutual Information (MI) scores. This method proved particularly advantageous in our particular scenario, where our dataset comprised a combination of linear and non-linear relationships that the MI technique could accurately capture. The ranking of features according to their MI scores provided a distinct perspective on which features could serve as the most significant predictors of the target variable. The model could be simplified by identifying and retaining solely the most informative features, thereby mitigating the potential for overfitting and enhancing interpretability.

The Pearson correlation coefficient is calculated to quantify the linear association between two continuous variables within the dataset (Obilor and Amadi, 2018). Moreover, principal component analysis (PCA) was used to reduce the dataset's dimensionality by retaining principal components that explained 95% of the variance. Based on PCA, 12 optimal features were selected. The selected features are: “A1_Score”, “A2_Score”, “A3_Score”, “A4_Score”, “A5_Score”, “A6_Score”, “A7_Score”, “A8_Score”, “A9_Score”, “A10_Score”, “ethnicity”, “contry_of_res”. These components are linear combinations of the original features, which pose challenges for direct interpretability. To address this, we projected the SHAP values of the principal components back into the original feature space using PCA loadings. This approach allowed us to identify the contribution of each original feature to the retained components and, by extension, to the model's predictions.

To ensure unbiased evaluation and generalizability of the proposed model, the data was split into two subsets: 80% for training and 20% for testing, using a random stratified sampling approach to maintain the class distribution in both subsets. The training data was further subjected to 5-fold cross-validation to validate the model's performance and tune hyperparameters. During cross-validation, the training data was divided into five folds, with four folds used for training and the remaining fold for validation in each iteration. This process was repeated five times, ensuring each fold was used once as the validation set. The final model was trained on the entire training set using the best hyperparameters obtained during cross-validation and evaluated on the independent test set. This approach mitigates the risk of overfitting and ensures reliable estimates of the model's performance on unseen data.

### 3.3 Stacked ensemble model

**Figure 3:**
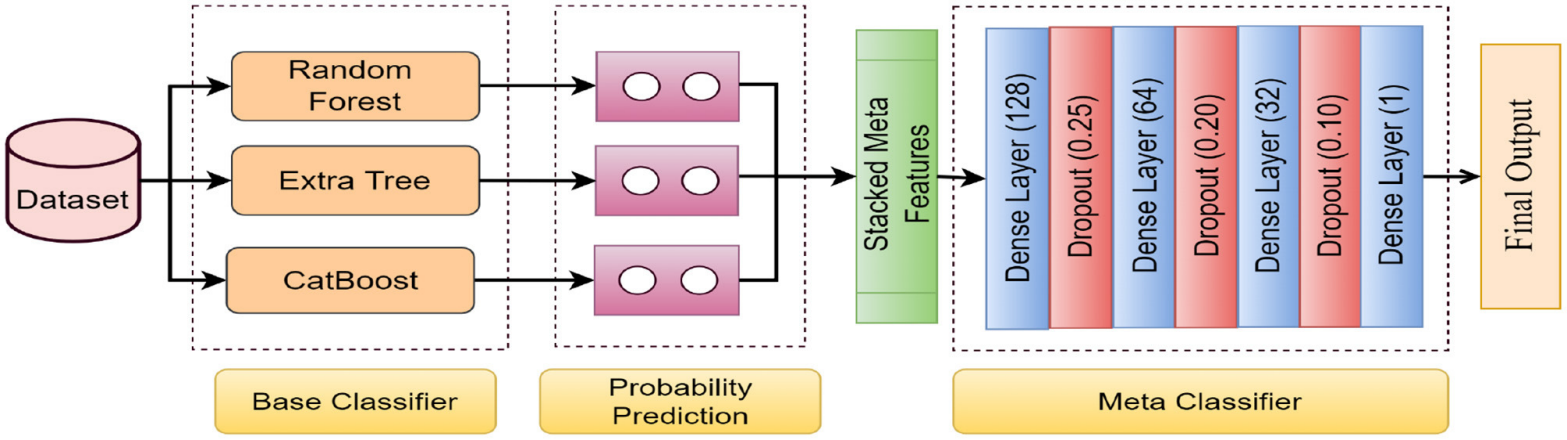
The architecture of the proposed stacked EM. The data is passed through the four base classifiers separately at first. Then, the base classifiers' predictions are stacked together to generate the meta-features. Finally, the meta-features are passed through the meta classifier to generate the final output.

Stacking, also referred to as stacked generalization using the ensemble method, operates on a straightforward principle: rather than relying on basic functions like the voting ensemble, all predictors' predictions are combined. One advantage of stacking is its ability to leverage the performance of several high-performing models in a classification or regression task, resulting in predictions that surpass the performance of any individual model within the ensemble (Sesmero et al., 2015; Naimi and Balzer, 2018). The primary objective is to incorporate the benefits of distinct and discrete models into the hybrid ensemble model while minimizing their drawbacks. The architecture of the proposed stacked EM is shown in in Figure 3.

#### 3.3.1 Base-classifier

##### 3.3.1.1 Random forest

Random forest is a frequently implemented supervised ML algorithm used to address classification and regression issues. The algorithm comprises numerous DTs, each of which processes a distinct subset of the dataset and calculates the mean to improve the prediction's precision. RF is an EL technique that reduces overfitting and outperforms a single DT by aggregating the results (Biau and Scornet, 2016).

##### 3.3.1.2 CatBoost

CatBoost (CB) (Prokhorenkova et al., 2018) is a gradient boost algorithm designed to efficiently handle categorical features. Incorporating innovative techniques to achieve high performance and robustness, particularly in scenarios with heterogeneous data types and large-scale datasets. CB minimizes a differentiable loss function L(*y*_*i*_, F(*x*_*i*_)), where *y*_*i*_ is the target variable, *F*(*x*_*i*_) is the predicted value for the *i*^*th*^ instance, and *x*_*i*_ is the characteristic vector. CB sequentially builds an ensemble of DTs to minimize the loss function.

##### 3.3.1.3 Extra trees

The ET algorithm, also known as Extremely Randomized Trees, is an EL method that belongs to the family of tree-based algorithms. The system initially generates many DTs during the training phase. Subsequently, it determines the output class based on the mean prediction (regression) or mode of the classes (classification) of the individual trees. The fundamental concept underlying the ET algorithm is the incorporation of randomization, which augments the model's variance to mitigate the risk of overfitting (Alsariera et al., 2020).

#### 3.3.2 Meta-learner

##### 3.3.2.1 Artificial Neural Network

A feed-forward Neural Network (FFN) is a type of network that creates a directed graph with nodes and edges. Data are transmitted along these edges from one node to the next without forming a cycle. The ANN is a variant of FFN with three or more layers: an input layer, one or more hidden layers, and an output layer. Researchers utilize a hyperparameter optimization approach to ascertain the optimal number of concealed layers for an ANN. The process of information transfer between layers does not consider previous values, and all neurons in each layer are interconnected, as supported by the sources (Goodfellow et al., 2016).

#### 3.3.3 Proposed ensemble model

In this study, we have proposed a deep EL framework that synergistically combines multiple base classifiers with an ANN-based metaclassifier. The overarching goal is to leverage the diverse strengths of various classifiers to enhance the model's predictive performance. In the base classifier part of the EM, three different classifiers are employed, each processing the input data and providing outputs that will be used to create meta-features for the meta-classifier.

Let the input dataset be represented as X=[*x*_1_, *x*_2_, ..., *x*_*n*_], where each *x*_*i*_ is a feature vector representing an individual sample, and n is the total number of samples. Correspondingly, the target labels are denoted by Y=[*y*_1_, *y*_2_, ..., *y*_*n*_], where each *y*_*i*_ is the binary class label associated with *x*_*i*_. Each base classifier *C*_*j*_ is trained on the dataset X with the goal of learning a mapping function, which predicts the probability that a given sample *x*_*i*_ belongs to the positive class. The training process involves optimizing the parameters of *C*_*j*_ to minimize the discrepancy between the predicted labels *y*^*i*^(*j*) and the actual labels *y*_*i*_.


(1) $$\begin{array}{l} F_{i}:X_{\text{train}}\to Y_{\text{predicted}}^{\left(i\right)} \end{array}$$

Upon training, each classifier *C*_*j*_ generates a predictive probability for each sample in the training set *X*_*train*_, validation set *X*_*val*_, and test set *X*_*t*_*est*. For a given sample *x*_*i*_, the output is a probability score $P_{\text{i}}^{\left(j\right)}$ indicating the likelihood that *x*_*i*_ belongs to the positive class, according to classifier *C*_*j*_. This can be formally represented as:


(2) $$\begin{array}{l} P_{i}^{\left(j\right)}=P\left(y_{i}=1|x_{i};\theta_{j}\right) \end{array}$$

which denotes the conditional probability that the label *y*_*i*_ is 1 given the feature vector *x*_*i*_ and the parameters θ_*j*_ of the classifier *C*_*j*_. For each sample, the predictive probabilities of all base classifiers are aggregated to form a new feature vector *x*_*meta*_, which serves as input to the meta-learner. The aggregation for a sample *x*_*i*_ across m base classifiers can be represented as:


(3) $$\begin{array}{l} x_{i_{\text{meta}}}=\left[p_{i}^{\left(1\right)},p_{i}^{\left(2\right)},\ldots ,p_{i}^{\left(m\right)}\right] \end{array}$$

where m is the number of base classifiers, the base classifiers effectively transform the original feature space into a meta-feature space of predictive probabilities. These meta-features, encapsulating the predictions from diverse algorithms, are then utilized by the meta-learner to make the final classification decision. This two-tier approach aims to capitalize on the strengths of individual classifiers and enhance overall predictive performance by synthesizing their predictions. In the EL framework, the meta-classifier operates as the second or final layer in the model hierarchy, synthesizing the outputs of the base classifiers to make a final prediction. The meta-classifier receives as input the meta-features *X*_*meta*_, composed of the predictive probabilities or decisions made by the base classifiers. For a given instance *x*_*i*_, the input to the meta-classifier can be represented as:


(4) $$\begin{array}{l} X_{\text{meta}}\left(i\right)=\left[p_{i}\left(1\right),p_{i}\left(2\right),\ldots ,p_{i}\left(m\right)\right] \end{array}$$

where $P_{\text{i}}^{\left(j\right)}$ is the probability that *x*_*i*_ belongs to the positive class as predicted by the *j*^*th*^ base classifier, and m is the total number of base classifiers.

The meta-classifier, denoted as *C*_meta_, is trained on this transformed dataset *X*_meta_ to learn a mapping function *f*_meta_:*X*_meta_→*Y* which aims to predict the final class label *y*_*i*_ for each instance *x*_*i*_. The function *f*_meta_ is optimized to minimize the discrepancy between its predictions ŷ^meta^ and the actual class labels *y*_*i*_. The output of the meta-classifier for each instance *x*_*i*_ is a final prediction ŷ^meta^, which is based on the aggregated insights from the base classifiers' predictions. This prediction can be a class label for classification tasks or a continuous value for regression tasks. For binary classification, the output can also be a probability score ${\hat{p}}^{\text{meta}}$ representing the likelihood that *x*_*i*_ belongs to the positive class.

For class labels:


$$\begin{array}{l} {ŷ}^{\text{meta}}=C_{\text{meta}}\left(X_{\text{meta}}\left(i\right)\right) \end{array}$$

For probability estimates:


$$\begin{array}{l} {\hat{p}}^{\text{meta}}=P\left(y_{i}=1|X_{\text{meta}}\left(i\right);\theta_{\text{meta}}\right) \end{array}$$

where θ_meta_ represents the parameters of the meta-classifier.

The first layer is a Dense layer with 128 neurons, using the ReLU activation function. It is set to receive input data corresponding to the meta-features generated by the base classifiers. After that, 2 hidden layers are used, having 64 and 32 nodes. A dropout layer was used after each layer, which helped reduce the overfitting issue. Dropout rate has been set to 25%. Lastly, an output layer with 1 node corresponded to 1 output class. The number of layers and nodes was set after much experimentation to find the best possible outcome. ReLu and Sigmoid have been used as the input and output activation functions. We have used Adam as the optimizer and Binary_Crossentropy as loss function. The learning rate was to 0.001 and number of epochs to 10.

## 4 Evaluation

### 4.1 Evaluation metrics

Several evaluation metrics have been utilized to evaluate the effectiveness of the proposed model, i.e., *Precision, Recall*, *F*_1_ *Score, Accuracy*, and *AUC-Score*.

### 4.2 Result analysis

**Figure 4:**
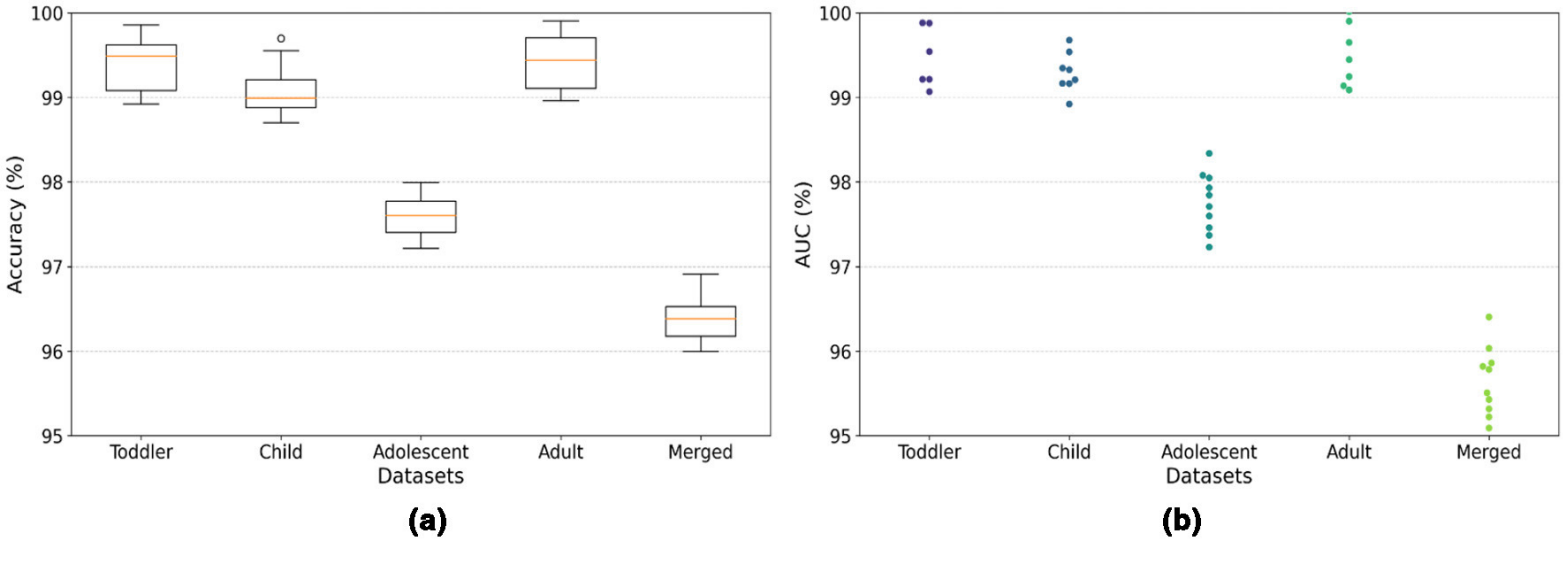
**(a)** Box-and-whisker plot of accuracy, and **(b)** swarm plot of AUC for all five datasets.

**Figure 5:**
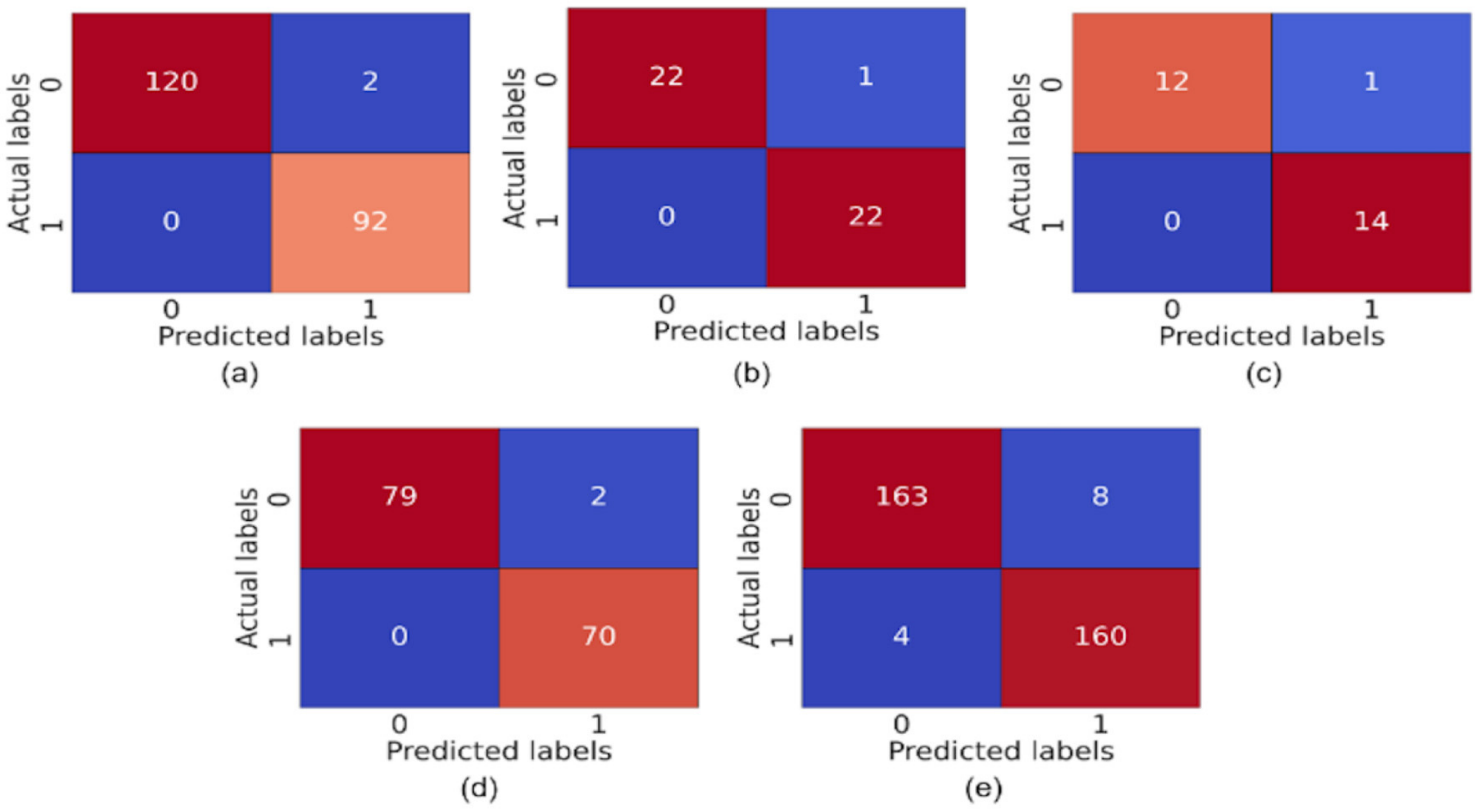
Confusion matrix of **(a)** toddler, **(b)** child, **(c)** adolescent, **(d)** adult, and **(e)** merged dataset.

The model demonstrates robust performance across various metrics, indicating its effectiveness in classifying ASD. The proposed model achieved an accuracy of 99.86%, 99.68%, 98.17%, 99.89%, and 96.96% in the Toddler, Child, Adolescent, Adult, and Merged datasets, respectively. Figures 4a, b present the box-and-whisker plot of accuracy and swarm plot of AUC for all five datasets used in this study. The confusion matrix of all these performance measures are shown in Figure 5. Table 1 presents the results obtained through the experiment of the proposed model. It can be deduced that the model can effectively identify a given sample as ASD or non-ASD.

**Table 1:** Results obtained from the proposed model.

**Dataset**	**Class**	**Precision**	**Recall**	**F1-score**	**Validation accuracy**	**Validation loss**	**Accuracy**	**AUC**
**1**	Class 0	99.97	100.00	99.93	99.95	0.0014	99.86	99.98
		Class 1	99.88	99.97					99.91				
**2**	Class 0	99.99	98.97	98.38	99.85	0.0412	99.68	99.89
		Class 1	99.99	98.29					98.21				
**3**	Class 0	99.99	98.38	98.89	99.11	0.0845	98.17	98.16
		Class 1	99.94	95.11					97.47				
**4**	Class 0	99.98	99.97	99.98	99.96	0.0036	99.89	99.99
		Class 1	99.99	99.97					99.98				
**5**	Class 0	98.15	97.68	97.87	98.74	0.0078	96.96	96.04
		Class 1	98.34	96.61					97.35				

The model achieved a perfect AUC score of 99.98%, indicating an exceptional ability to distinguish between ASD and non-ASD cases among toddlers. This result is particularly significant given the challenges of early diagnosis of ASD and the importance of timely intervention. With an AUC of 99.89%, the model demonstrated near-perfect performance in the Child dataset. This high score underscores the model's robustness and its potential utility in supporting clinicians and caregivers in the early detection of ASD in children. The model achieved an AUC of 98.16%, showcasing its strong discriminative power in identifying ASD among adolescents. This highlights the applicability of the model in a broad age range, addressing the varying presentation of ASD symptoms as children grow. Mirroring its success with the Toddler dataset, the model once again achieved a perfect AUC score of 99.99% for the Adult dataset. This remarkable consistency across the developmental spectrum emphasizes the model's comprehensive applicability and reliability in ASD screening for all age groups. The performance of the model on the Merged dataset, which amalgamates data across all age categories, resulted in an AUC of 96.04%. While slightly lower than the age-specific datasets, this score is still exceptionally high. It illustrates the model's effectiveness in handling a diverse and complex dataset that reflects the broad variability in ASD presentations across different ages.

**Figure 6:**
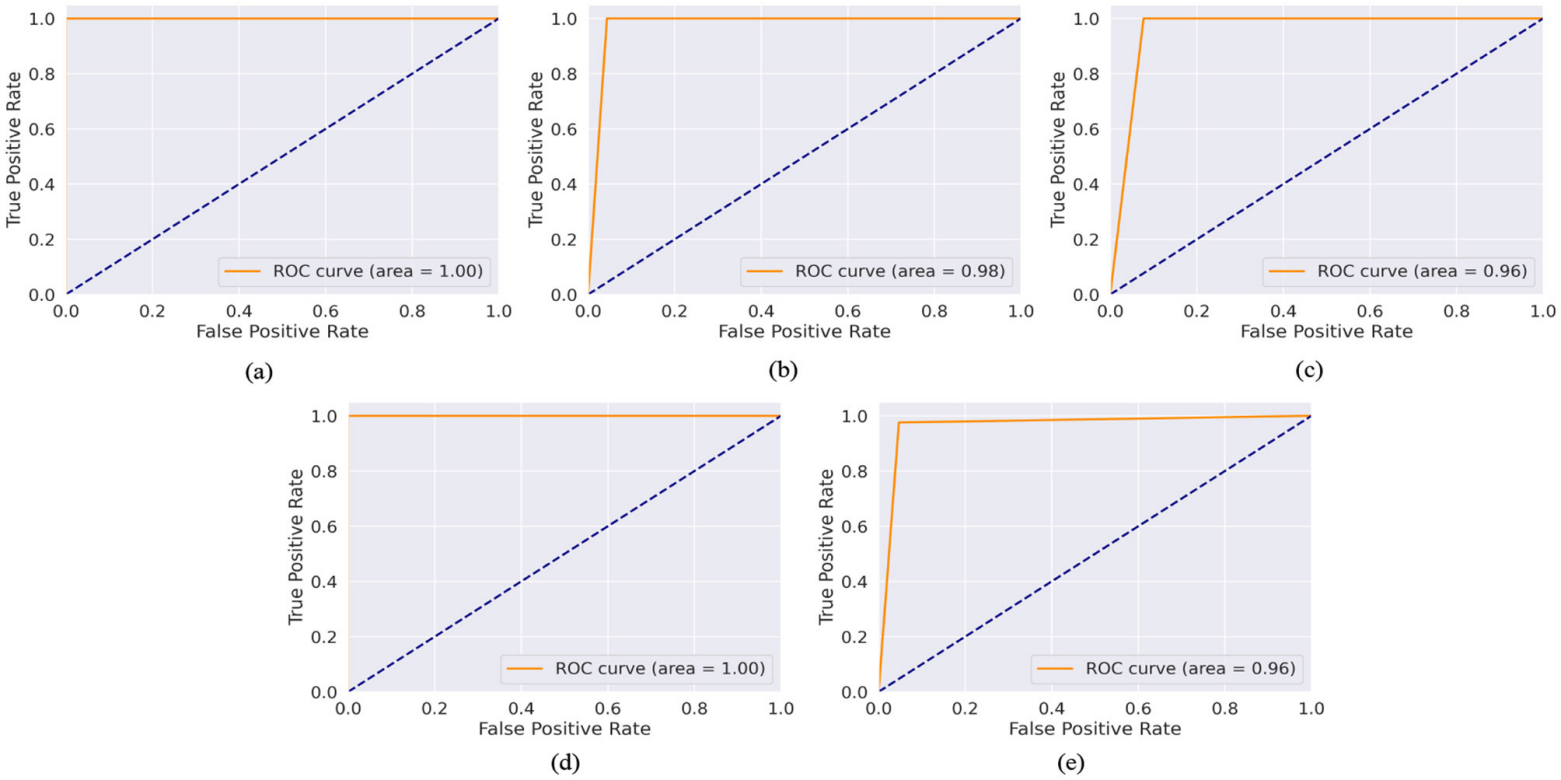
AUC-ROC curve of **(a)** toddler, **(b)** child, **(c)** adolescent, **(d)** adult, and **(e)** merged dataset.

The ROC-AUC graphs for the Toddler, Child, Adolescent, Adult, and Merged datasets are depicted in Figures 6a–e respectively. The ROC curve is a plot with the TPR on the y-axis and the FPR on the x-axis at various threshold settings. Figure 6a depicts a ROC curve with an AUC of 1.00. This value quantifies the overall ability of the model to discriminate between the positive and negative classes. The TPR (sensitivity) is constant at 1.0 across all levels of the FPR. This means that the model correctly identifies all positive cases regardless of the number of false positives. The FPR changes from 0.0 to 1.0 without affecting the TPR, which remains perfect throughout. The ROC curve is a horizontal line at the top of the plot area, indicating a perfect classifier. The AUC of 1.00 confirms this, as it suggests that the model has a perfect separability measure, meaning it can distinguish between positive and negative classes without error. Figure 6d is similar as Figure 6a. In Figure 6b ROC-AUC value is 0.98, which is very close to 1. The curve approaches the left-hand side and the top of the ROC space, indicating high sensitivity (TPR) and high specificity (low FPR). The model maintains a high TPR even as the FPR increases slightly, showing that the classifier is robust across different threshold settings. In Figures 6c, e ROC-AUC value is 0.96, which is very close to 1. The curve approaches the left-hand side and the top of the ROC space, indicating high sensitivity (TPR) and high specificity (low FPR). The model maintains a high TPR even as the FPR increases slightly, showing that the classifier is robust across different threshold settings.

### 4.3 Impact of feature selection

The PCA feature reduction method enhances the model's assessment precision. Using PCA reduces the number of parameters by exclusively selecting the significant features that account for the explained maximum variance. By implementing this method, the quantity of parameters is drastically reduced. Additionally, the accuracy of testing is marginally enhanced by excluding non-significant features. The comparison of test accuracy performance before and after PCA implementation is presented in Table 2 (feature importance bar graphs, Supplementary Figures S2–S6).

**Table 2:** Performance of the proposed model before and after feature selection.

**Dataset**	**Before feature selection**					**After feature selection**				
	**Acc**	**Pre**	**Rec**	**F1**	**AUC**	**Acc**	**Pre**	**Rec**	**F1**	**AUC**
Toddler	98.12	99.04	97.26	98.58	98.03	99.86	99.92	99.98	99.92	100.00
Child	96.58	98.29	95.27	96.88	97.63	99.68	99.99	98.28	99.27	99.89
Adolescent	96.69	95.97	96.58	95.98	96.89	98.17	99.97	97.07	98.02	98.16
Adult	98.33	97.98	99.06	98.65	97.48	99.89	99.98	99.97	99.98	100.00
Merged	92.88	93.25	91.32	92.47	93.58	96.96	98.25	97.13	97.59	96.04

### 4.4 Impact of balancing class

The bias can result in poor predictive accuracy for the minority class, which is often of greater interest in medical and psychological research, including the diagnosis of ASD. Using the safe-level-SMOTE to balance classes in this study addresses the inherent challenges of imbalanced datasets. Safe-level-SMOTE, an advanced oversampling technique, generates synthetic samples for the minority class based on “safety” levels, which considers the data distribution to create more realistic and representative samples.

Table 3 presents the performance of the proposed model before and after applying Safe-level-SMOTE in the datasets. This enhancement is attributed to the algorithm's ability to mitigate the bias toward the majority class by enriching the dataset with synthetic, yet plausible, minority-class samples. This balanced class distribution allows for a more equitable learning environment, where the classifier can learn to recognize patterns and characteristics of both classes without being overwhelmed by the majority class.

**Table 3:** Performance of the proposed model before and after addressing class imbalance.

**Dataset**	**Before safe-level SMOTE**					**After safe-level SMOTE**				
	**Acc**	**Pre**	**Rec**	**F1**	**AUC**	**Acc**	**Pre**	**Rec**	**F1**	**AUC**
Toddler	97.53	99.33	97.51	98.51	97.73	99.86	99.92	99.98	99.92	100.00
Child	97.43	98.64	97.46	96.88	98.90	99.68	99.99	98.28	99.27	99.89
Adolescent	97.39	96.67	97.58	96.78	96.94	98.17	99.97	97.07	98.02	98.16
Adult	97.68	98.59	98.54	98.59	97.77	99.89	99.98	99.97	99.98	100.00
Merged	94.72	94.15	92.37	93.67	94.78	96.96	98.25	97.13	97.59	96.04

### 4.5 Statistical analysis

To validate the statistical significance of the proposed model's performance over baseline machine learning models, a Wilcoxon signed-rank test was conducted. This test compared the proposed model against various baseline ML models. The test was applied to two key metrics: Accuracy, and F1-Score, across five datasets. The results, summarized in Table 4, indicate that the p-values for all comparisons are above the significance threshold (0.05). This suggests that while the proposed model consistently achieved high performance across all metrics and datasets, the improvements were not statistically significant compared to the baseline models.

**Table 4:** Wilcoxon signed-rank test results for accuracy comparing the proposed model's performance with baseline machine learning models across five datasets.

**Dataset**	**SVM**	**LR**	**DT**	**RF**	**GB**	**XGB**	**CB**	**ET**	**KNN**	**NB**	**ANN**	**LDA**
Toddler	0.822	1.000	0.118	0.625	0.445	0.605	0.482	0.750	0.220	0.060	0.568	0.215
Children	0.765	0.950	0.102	0.530	0.410	0.589	0.460	0.701	0.200	0.054	0.520	0.198
Adolescent	0.740	0.880	0.150	0.600	0.455	0.620	0.500	0.770	0.210	0.070	0.550	0.210
Adult	0.700	0.910	0.130	0.610	0.420	0.590	0.470	0.740	0.180	0.050	0.530	0.205
Merged	0.750	0.930	0.140	0.620	0.430	0.610	0.480	0.720	0.190	0.055	0.540	0.210

### 4.6 Handling overfitting

Overfitting, where a model performs well on the training data but poorly on unseen data, is a critical challenge for machine learning models. In this study, we adopted multiple strategies to mitigate overfitting and ensure the generalizability of the proposed ensemble learning framework. First, dimensionality reduction techniques, including PCA and MI-based feature selection, were applied to remove redundant and irrelevant features. This streamlined the model by retaining only the most informative features, reducing the risk of learning noise from the data. Second, within the ANN meta-classifier, dropout layers were employed with a dropout rate of 25%. Dropout is an effective regularization method that prevents overfitting by randomly deactivating neurons during training, which forces the network to learn more generalized patterns. Additionally, we implemented k-fold cross-validation (k = 5) to validate the model's robustness. Cross-validation splits the data into multiple training and validation subsets, ensuring the model is evaluated across different data partitions, which reduces variance and improves generalization to unseen data. MCD was applied during inference to estimate uncertainty in predictions to ensure reliability further. MCD helps evaluate the consistency and confidence of the model, enabling the detection of potential overfitting by analyzing output variations on test data. Finally, Safe-Level SMOTE addressed the class imbalance in the datasets, enhancing the model's ability to learn representative patterns from the minority class without overfitting to majority class samples. These combined techniques ensure that the proposed model remains robust, reliable, and free from overfitting, thus enhancing its applicability in real-world scenarios.

### 4.7 Comparison with existing works

In this research paper, we implemented an innovative methodology for the screening of ASD utilizing a stacked ensemble-based model. This model combines several ML algorithms to analyze the data obtained from the ASD screening questionnaires. The method we have developed is notable for its resilience and precision, supported by the exhaustive comparative analysis provided in the Table 5. The research is situated within the context of prior investigations that have sought to improve the precision and dependability of ASD screening instruments. In brief, the comparative analysis highlights the progress that our stacked EM contributes to ASD screening. The proposed work enhances the continuous endeavor to develop ASD screening tools that are clinically valuable, dependable, and accurate by resolving certain constraints identified in prior studies, including overfitting and the trade-off between recall and precision. The results of our research support the incorporation of advanced ML methods into the evaluation of ASD, which has great potential to advance the detection and treatment of individuals on the spectrum.

**Table 5:** Comparison with existing works on the used datasets.

**Dataset**	**Reference**	**Acc**	**Pre**	**Rec**	**F1**	**AUC**
Toddler	Uddin et al., 2023	99.85	**1.00**	**1.00**	99.85	99.85
		Akter et al., 2021a	98.77	-	-	-	**99.98**
		Bala et al., 2022	97.82	-	-	97.8	99.7
		Hasan et al., 2021	99.25	99.89	98.45	99.1	
		Priyadarshini, 2023	99.64	96	94	91	-
		Vakadkar et al., 2021	-	-	-	98.00	-
	Mohanty et al., 2021	85.24	-	-	82.00	-
	Proposed	**99.86**	99.94	99.86	**99.90**	99.95
Child	Talabani and Engin, 2018	92.26	88.09	96.52	-	-
		Abitha et al., 2022	94.1	-	-	-	-
		Omar et al., 2019	92.26	-	-	-	-
		Haroon and Padma, 2022	95.5	98	97	96	-
		Kamma et al., 2022	95.82	-	-	-	-
		Gupta et al., 2022	-	92.59	97.09	94.71	-
		Thabtah, 2019	97.80	-	98	-	-
		Bala et al., 2022	99.61	-	-	**99.60**	99.60
		Akter et al., 2019	97.20	-	-	-	99.89
		Mohanty et al., 2021	84.21	-	-	84.21	-
	Garg et al., 2022	98.00	-	-	-	-
	Hasan et al., 2021	97.95	96.16	97.72	97.02	99.73
	Proposed	**99.68**	**99.97**	**99.03**	99.58	**99.89**
Adolescent	Talabani and Engin, 2018	93.78	89.85	98.4	-	-
		Omar et al., 2019	93.78	-		-	-
		Thabtah, 2019	94.23	-	92.20	-	-
		Kamma et al., 2022	95.82	-	-	-	-
		Gupta et al., 2022	-	93.25	74.15	84.21	-
		Akter et al., 2019	93.89	-	-	-	**98.61**
		Bala et al., 2022	95.87	-	-	95.90	99.00
		Mohanty et al., 2021	85.71	-	-	88.52	-
	Hasan et al., 2021	97.12	97.25	**97.36**	97.69	99.72
	Proposed	**98.17**	**99.52**	97.18	**98.46**	98.16
Adults	Priyadarshini, 2023	98.89	94	91	93	
		Shuvo et al., 2019	95.71	-	85.71	-	-
		Kamma et al., 2022	95.82	-	-	-	-
		Gupta et al., 2022	-	97.46	91.27	94.26	-
		(Talabani and Engin, 2018)	96.91	90.07	96.87	-	-
		Akter et al., 2019	98.36	-	-	-	99.95
		Omar et al., 2019	97.10	-	-	-	-
		Abitha et al., 2022	98	-	-	-	-
		Bala et al., 2022	99.82	-	-	99.90	99.80
		Thabtah, 2019	99.85	-	99.90	-	-
		Mohanty et al., 2021	89.26	-	-	85.39	-
	Hasan et al., 2021	99.03	98.16	**100.00**	99.11	**99.99**
	Proposed	**99.89**	**99.98**	99.92	99.95	**99.99**

The bold values indicate the highest results obtained among all.

## 5 Explainable AI

SHapley Additive exPlanations (SHAP) is an innovative method within the domain of XAI that provides valuable insights into the results produced by ML models (Lundberg and Lee, 2017). The model-agnostic nature of SHAP is one of its assets. This feature enables XAI to employ diverse models, such as ensemble methods such as RFs and complex architectures like ANNs. SHAP focuses primarily on local explanations; however, by aggregating these explanations, one can obtain global insights regarding the model. This facilitates comprehension of the model's overall behavior, including the features that exert the greatest influence and their interrelationships. SHAP ensures explanations remain consistent; if a model undergoes modification resulting in an increase or maintenance of a feature's contribution, its SHAP value will not diminish. This guarantees that the explanations accurately represent the model's behavior.

Several stages are required to implement SHAP in a binary classification task utilizing a stacked EM consisting of multiple ML models as the base classifier and an ANN as the meta-classifier. Predictions were initially generated by each of the fundamental classifiers, namely the RF, ET, and CB. For each class, these predictions are presented as probability distributions. Subsequently, the predictions generated by the base classifiers were employed as meta-features for the ANN meta-classifier. Each feature in the intermediate dataset corresponds to the output of one of the base models. After the meta-classifier has been trained, its predictions are interpreted using SHAP. Being model-independent and compatible with ANNs, SHAP may be implemented directly on the meta-classifier. Currently, the SHAP values for the predictions produced by the meta-classifier can be generated. The magnitude to which the final decision for each class was influenced by the output (now a meta-feature) of each base classifier will be denoted by these values. A SHAP summary plot was utilized to provide clarification.

SHAP was implemented individually on each base classifier to ascertain how the input features impact their respective predictions. This dual-level explanation of the stacked EM (at both the base classifier and meta-classifier levels) provides a comprehensive understanding of the model. Furthermore, SHAP has been used for both local and global interpretation. For each specific class, a summary plot has been generated in local interpretation. Four graphs, one for each of the four classes, have been produced in total for the classifier. A summary plot has been produced for global interpretation by aggregating SHAP values. This plot provides valuable insights into the model's overall behavior.

**Figure 7:**
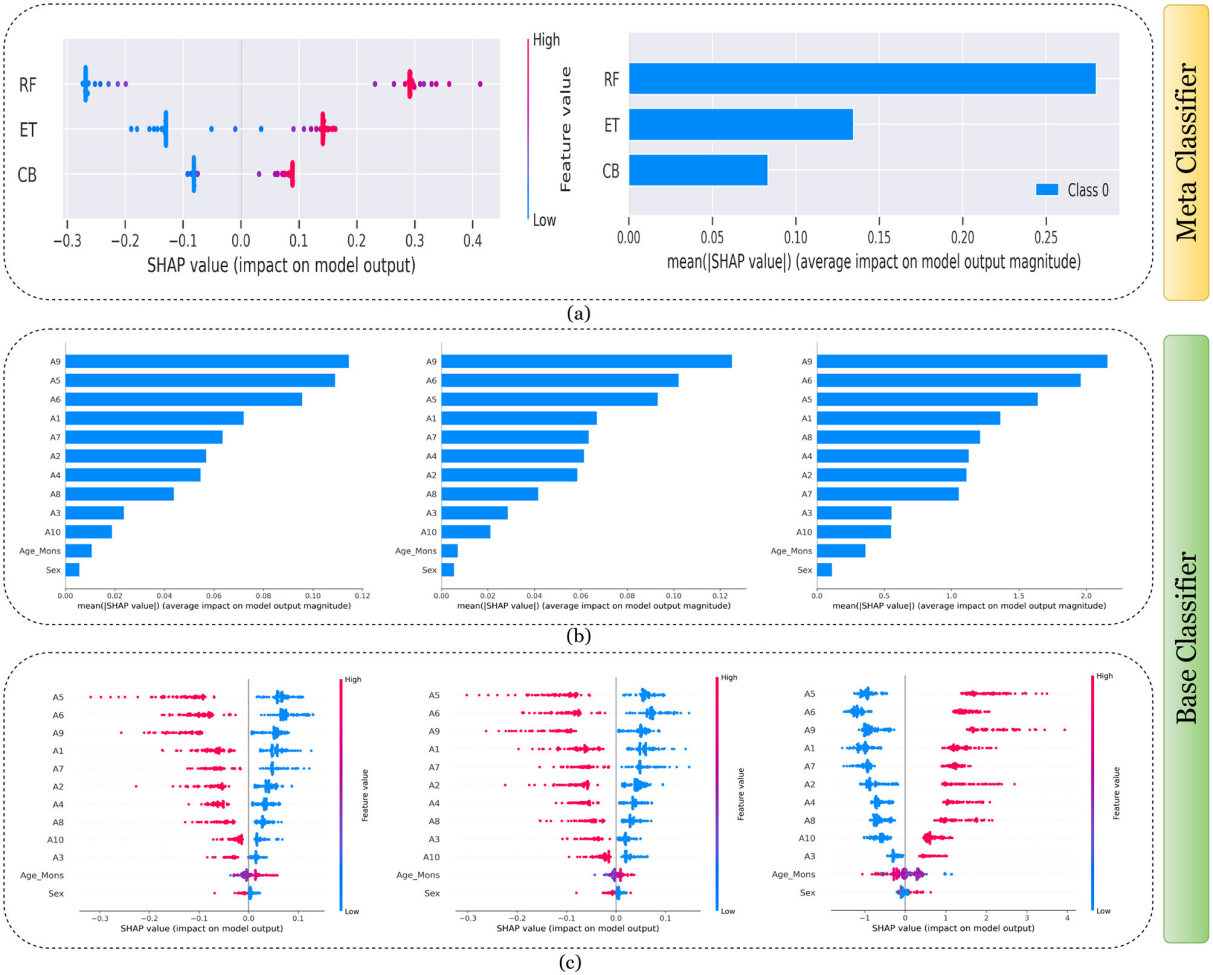
Explanation of models using SHAP on Toddler dataset. **(a)** Explaining meta-classifier using dot plot (left), and summary plot (right). Explanation of base classifiers, RF (left), ET (middle), CB (right) using **(b)** summary plot, and **(c)** dot plot.

**Figure 8:**
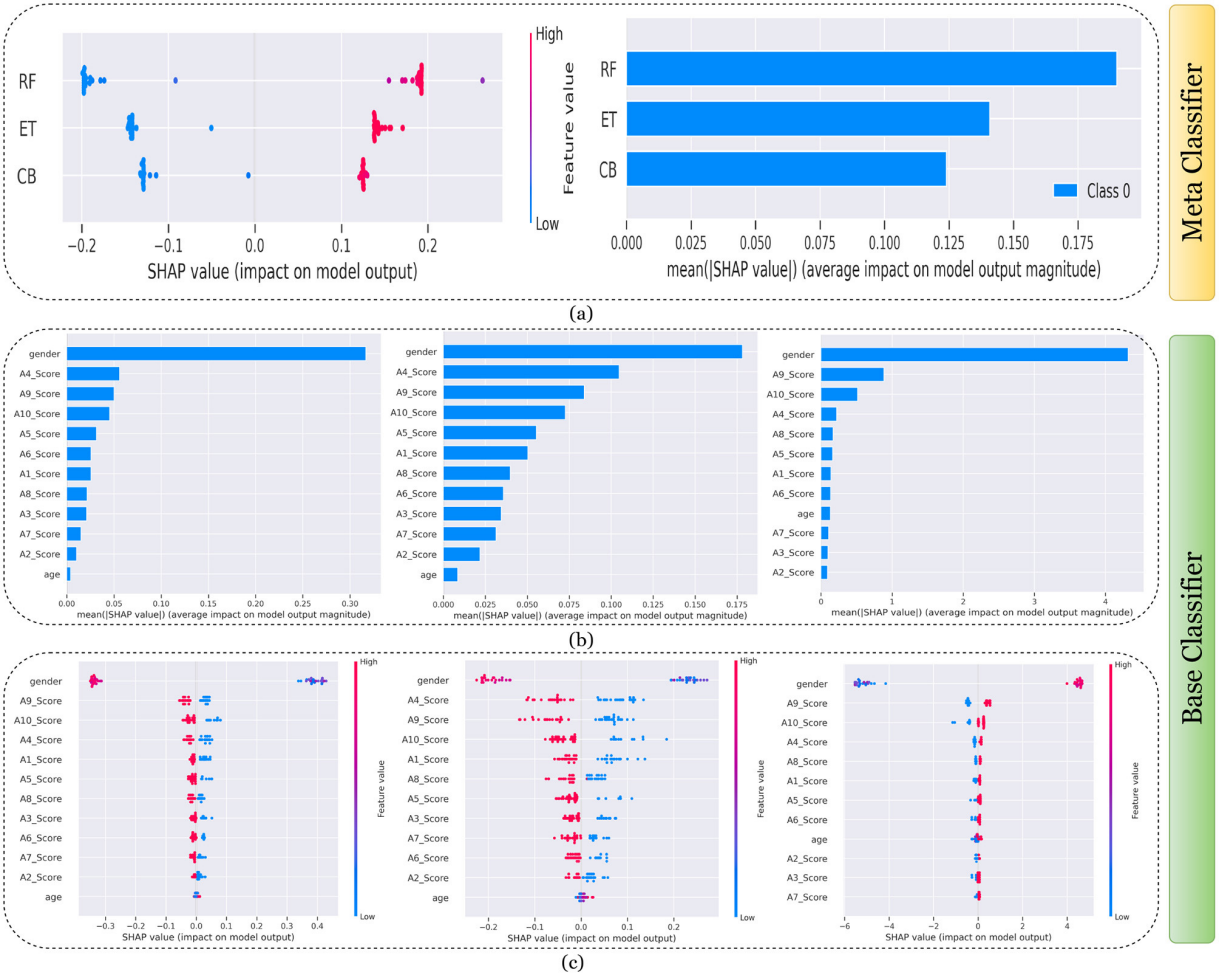
Explanation of models using SHAP on Child dataset. **(a)** Explaining meta-classifier using dot plot (left), and summary plot (right). Explanation of base classifiers, RF (left), ET (middle), CB (right) using **(b)** summary plot, and **(c)** dot plot.

**Figure 9:**
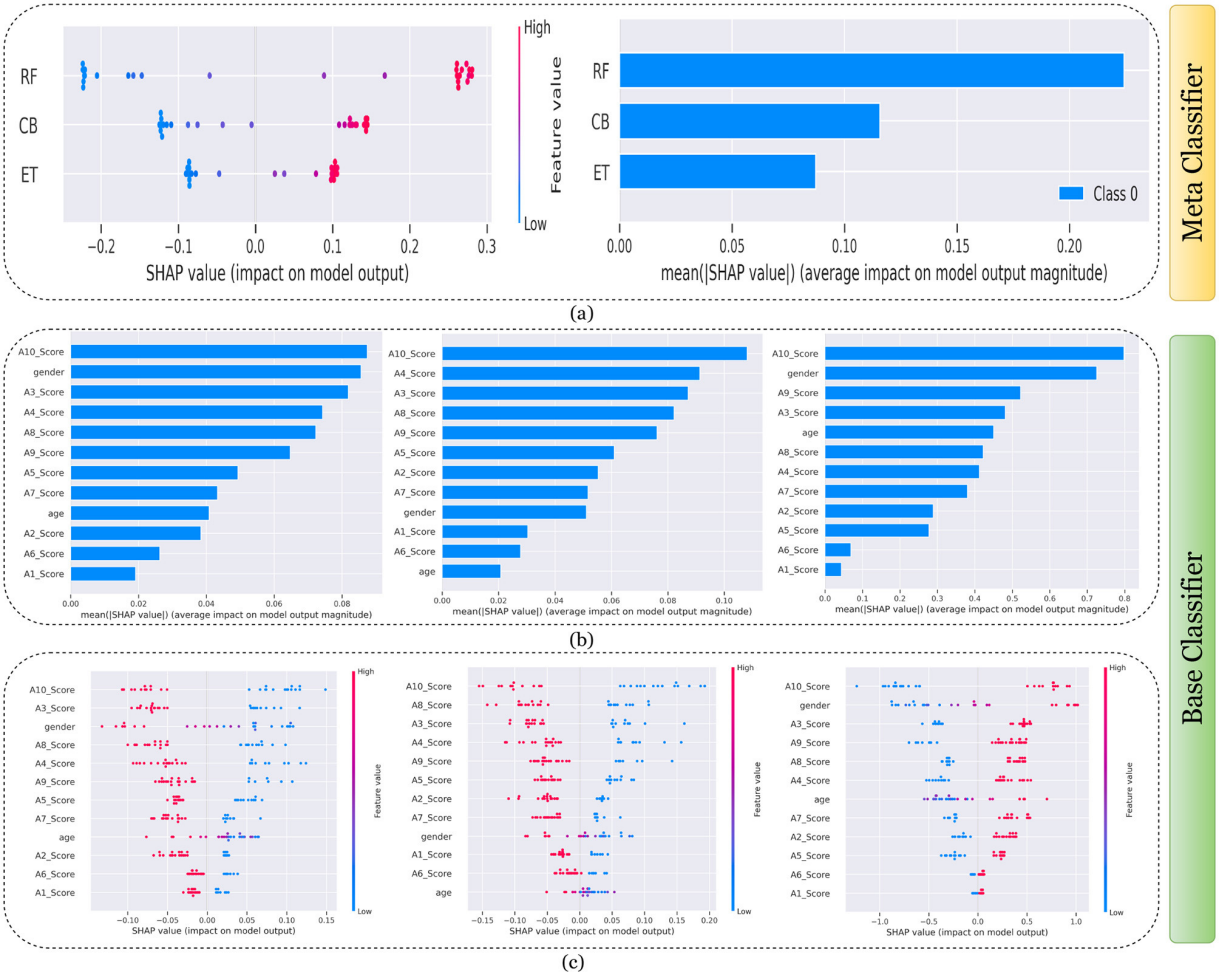
Explanation of models using SHAP on Adolescent dataset. **(a)** Explaining meta-classifier using dot plot (left), and summary plot (right). Explanation of base classifiers, RF (left), ET (middle), CB (right) using **(b)** summary plot, and **(c)** dot plot.

**Figure 10:**
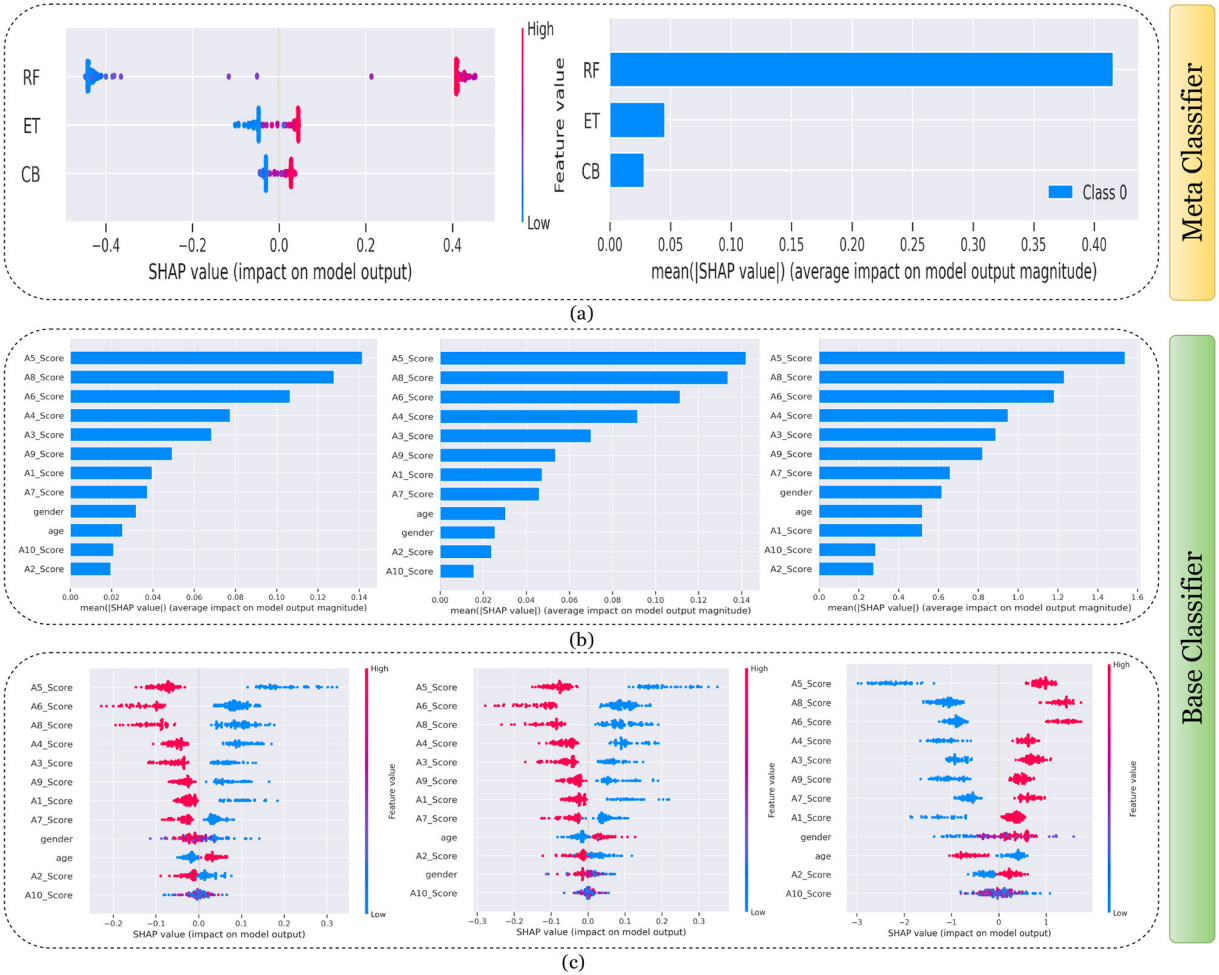
Explanation of models using SHAP on Adult dataset. **(a)** Explaining meta-classifier using dot plot (left), and summary plot (right). Explanation of base classifiers, RF (left), ET (middle), CB (right) using **(b)** summary plot, and **(c)** dot plot.

**Figure 11:**
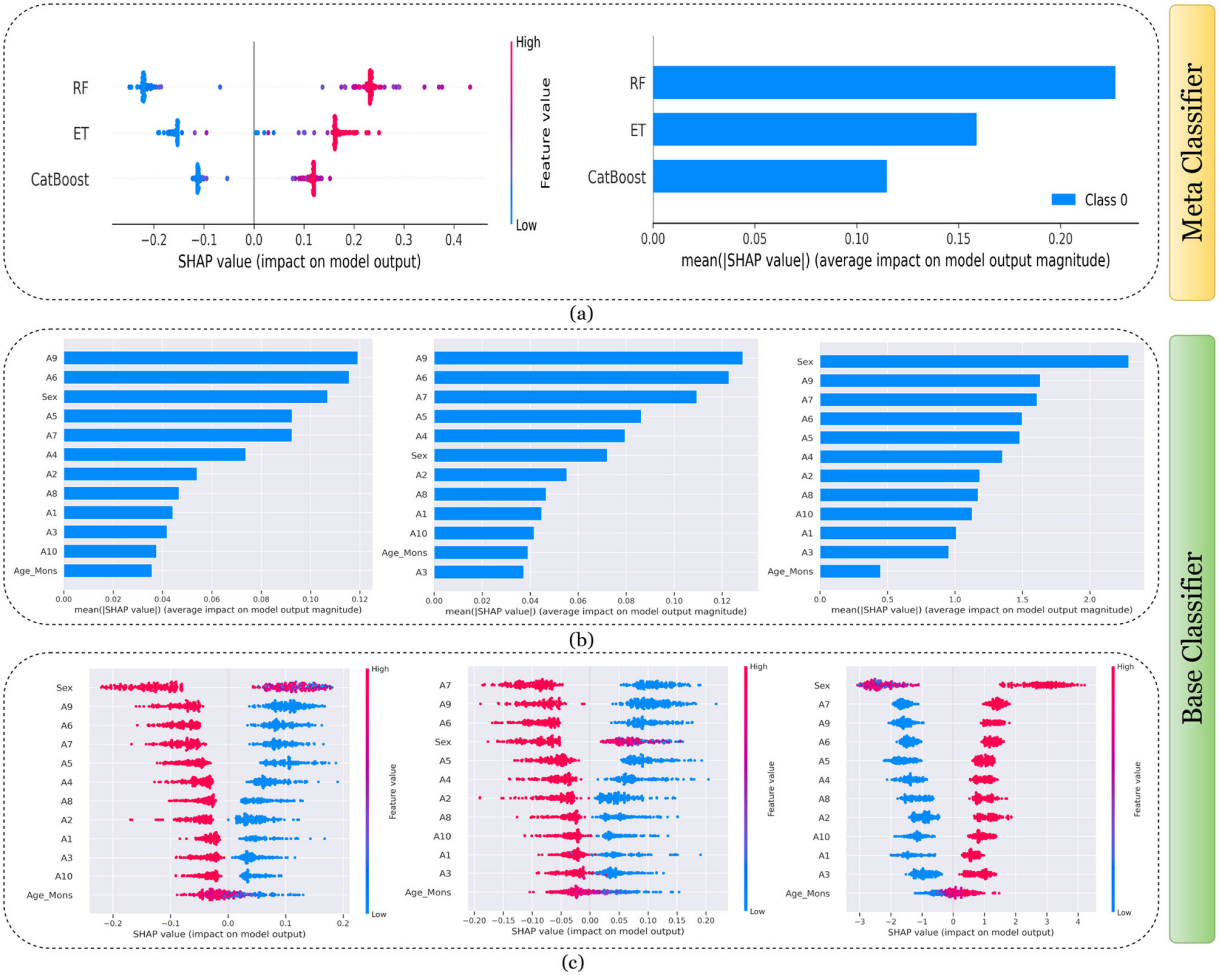
Explanation of models using SHAP on Merged dataset. **(a)** Explaining meta-classifier using dot plot (left), and summary plot (right). Explanation of base classifiers, RF (left), ET (middle), CB (right) using **(b)** summary plot, and **(c)** dot plot.

Figures 7–11 presents the explanations generated using SHAP on both meth classifier and base classifier for Toddler, Child, Adolescent, Adult, and Merged dataset respectively. Each figure is sepearted in two major parts that includes explaining the meta classifier using classwise summary plot (left), and overall summary plot (right), and then explaining the base classifiers utilizing overall summary plot (middle) and classwise summary plot (below). For example, a classwise summary plot (left) and overall summary plot(right) generated from meta classifier for Toddler dataset has been provided in Figure 7a. From the classwise summary plot (left), it can be observed that most SHAP values are centered around zero but show a spread on both the negative and positive sides, indicating that features both positively and negatively influence the model's output for RF model.

The ET model's SHAP values show a similar pattern to those of the RF model, with a distribution around zero and a spread to both sides, again suggesting a mix of positive and negative feature impacts, while the CB model's SHAP values are more concentrated around zero compared to the RF and ET models, with fewer extreme positive or negative values. This suggests that individual features may have a more uniform influence on the CB model's output. The presence of SHAP values above and below zero across the models indicates that features increase and decrease the likelihood of a positive class prediction. Meanwhile, in the classwise summary plot (left), SHAP values quantify the impact of features to the model's prediction, and the mean provides an overall measure of the different features' impact on the model's output. It can be observed that the RF model has a greater mean, indicating that, on average, its features have a substantial influence on the model output. The ET model has the second-highest mean, which is slightly less than that of the RF model, suggesting that its features have a strong but slightly lesser impact on model output than the RF model. The CB model has the lowest mean value among the three models, indicating that its features, on average, have less impact on the model output. The term “Class 0” in the legend suggests that these SHAP values are associated with the impact on a specific class in a classification problem, likely the negative class if we assume a binary classification task.

Findings from this chart imply that the RF model relies more heavily on individual features for making predictions or it has a few features with powerful impacts. In contrast, the CB model's predictions seem to be influenced less by individual features or have a more distributed influence across features. This could reflect differences in how the models handle feature interactions or their inherent algorithmic biases. Similarly, the classwise summary plot(middle) and overall summary plot (below) generated from the base classifier for the Toddler dataset have been provided in Figures 7b, c. In Figures 7b, c the predictions from RF (left), ET (middle), and CB (right) is provided. Figure 7b is a horizontal bar chart depicting the mean values for various features in a predictive model generated from RF (left), ET (middle), and CB (below). Figure 7c shows a detailed SHAP value scatter plot for various features in the base classifiers RF (left), ET (middle), and CB (below). SHAP values depict the impact of a given feature on the model's output for a prediction, with positive values indicating an increase in the likelihood of a particular outcome and negative values indicating a decrease. Similarly, Figures 8–11 can be interpreted to find the explanation provided by the models on the datasets.

The SHAP analysis provides critical insights into the significance of various features in predicting ASD across different age groups. The analysis highlights how feature importance shifts with developmental stages, reflecting the evolving nature of ASD markers over time. For example, A8, a behavioral trait, is less influential in toddlers but becomes a pivotal feature in adults, likely due to its association with advanced cognitive and social functions, such as abstract reasoning, self-awareness, and complex social interactions. These traits typically emerge in later developmental stages, making A8 more relevant in understanding ASD in adults. In contrast, features like A1 and A3 dominate the toddler dataset, as they are linked to early developmental markers such as sensory processing, responsiveness to stimuli, and essential social engagement, which are critical indicators of ASD at a younger age.

In children and adolescents, features such as A9 and A10 gain prominence, suggesting that adaptive behaviors and developmental milestones increase as children age and begin navigating more structured environments like school and peer interactions. The adolescent dataset further emphasizes features like A3 and A8, reflecting the developmental emergence of independence and higher-order cognitive abilities that are relevant during this transitional stage. For adults, behavioral and cognitive traits, represented by features like A8, become more critical as they pertain to advanced social, occupational, and emotional functioning, which are often areas of challenge for adults with ASD. The merged dataset presents a balanced representation of features, such as A1, A8, and A9, broadly significant across all age groups.

These variations underscore the dynamic nature of ASD manifestations and the necessity for diagnostic models tailored to specific age groups as the traits and behaviors associated with ASD evolve significantly over time. For instance, toddlers might display ASD-related traits primarily through sensory responses and early social behaviors, while adults may exhibit challenges in abstract reasoning, emotional regulation, and nuanced social interactions. This contextual understanding of feature importance across developmental stages provides actionable insights for clinicians and researchers and reinforces the importance of considering age-specific diagnostic markers.

## 6 Uncertainty analysis

UA is a technique utilized in the domain of ANNs to quantify a network's confidence level in its predictions (Bachstein, 2019). Neural networks, particularly DL models, are often regarded as opaque models that generate predictions without disclosing their level of certainty. To address this concern, the UA incorporates a confidence level into predictions. This notion is of notable significance in domains where decisions based on model predictions entail substantial implications. The Monte Carlo dropout (MCD) method (Gal and Ghahramani, 2016) is a technique used to quantify uncertainty in DL models. Estimating model uncertainty is a computationally efficient and practicable process that holds significant importance in numerous applications where confidence in model predictions is critical for decision-making. Initially, dropout was implemented in ANNs as a regularization technique to avert overfitting (Ahmed et al., 2023). MCD can be utilized to detect instances in which the predictions generated by the model are deemed unreliable. We have utilized MCD inference on individual samples, a calibration curve, variance analysis, and standard deviation analysis.

**Figure 12:**
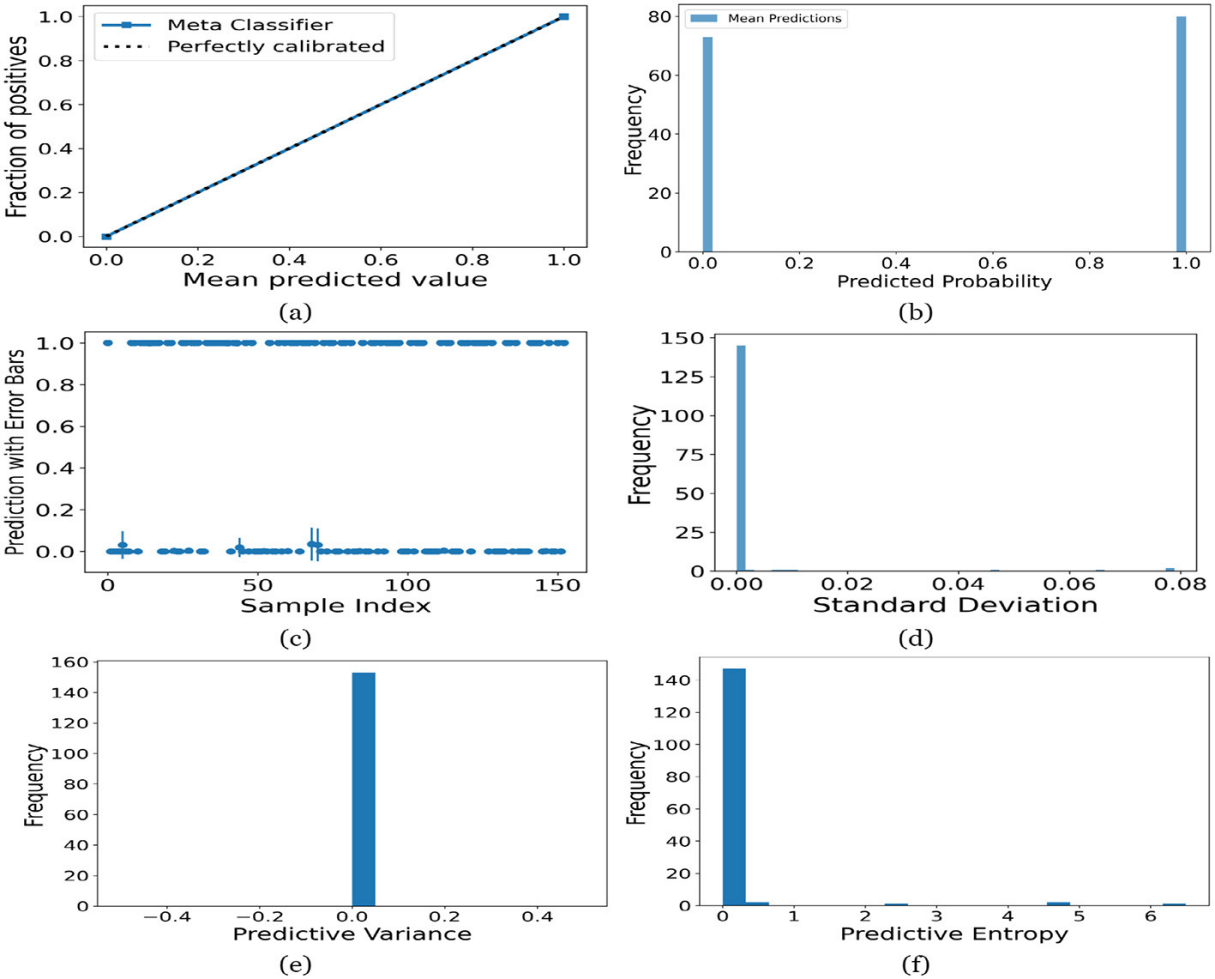
UA on Toddler dataset utilizing **(a)** calibration curve, **(b)** mean probability bar graph, **(c)** predictive error bar graph, **(d)** predictive standard deviation, **(e)** predictive variance, and **(f)** predictive entropy.

**Figure 13:**
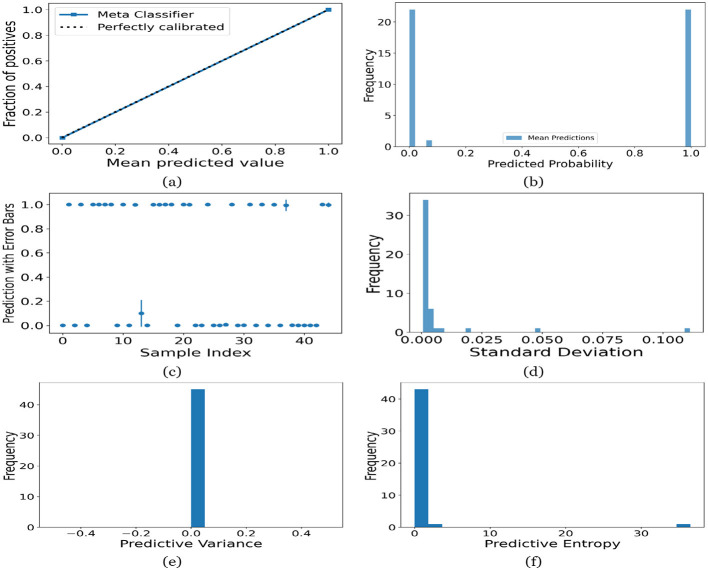
UA on Child dataset utilizing **(a)** calibration curve, **(b)** mean probability bar graph, **(c)** predictive error bar graph, **(d)** predictive standard deviation, **(e)** predictive variance, and **(f)** predictive entropy.

**Figure 14:**
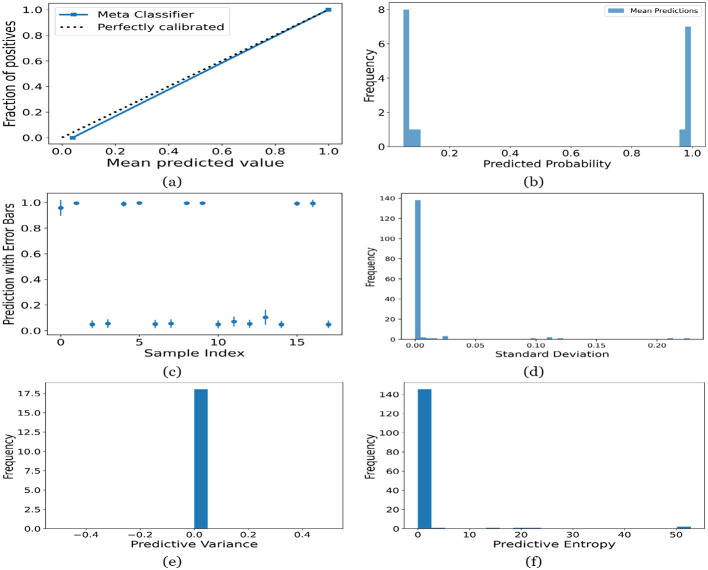
UA on Adolescent dataset utilizing **(a)** calibration curve, **(b)** mean probability bar graph, **(c)** predictive error bar graph, **(d)** predictive standard deviation, **(e)** predictive variance, and **(f)** predictive entropy.

**Figure 15:**
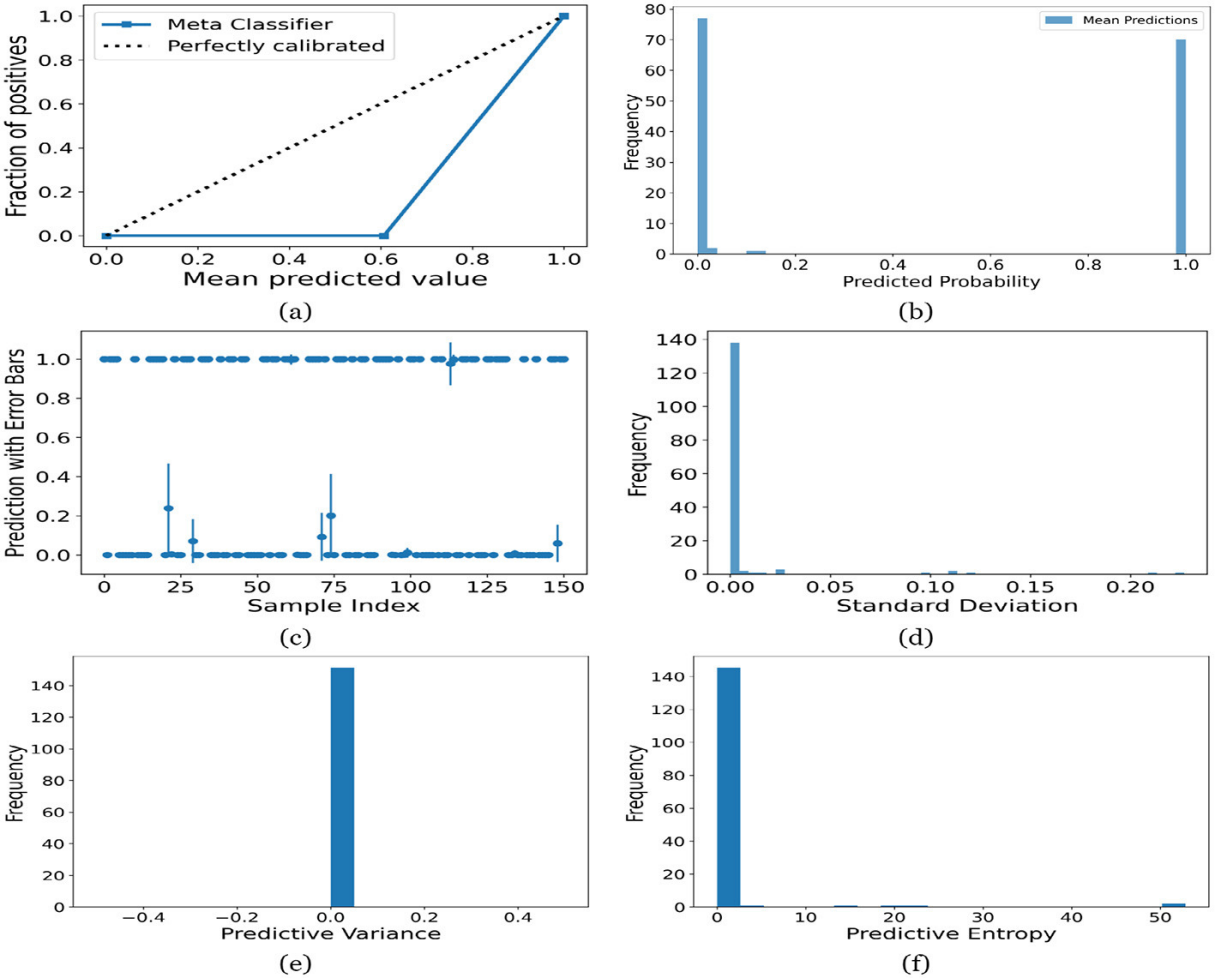
UA on Adult dataset utilizing **(a)** calibration curve, **(b)** mean probability bar graph, **(c)** predictive error bar graph, **(d)** predictive standard deviation, **(e)** predictive variance, and **(f)** predictive entropy.

**Figure 16:**
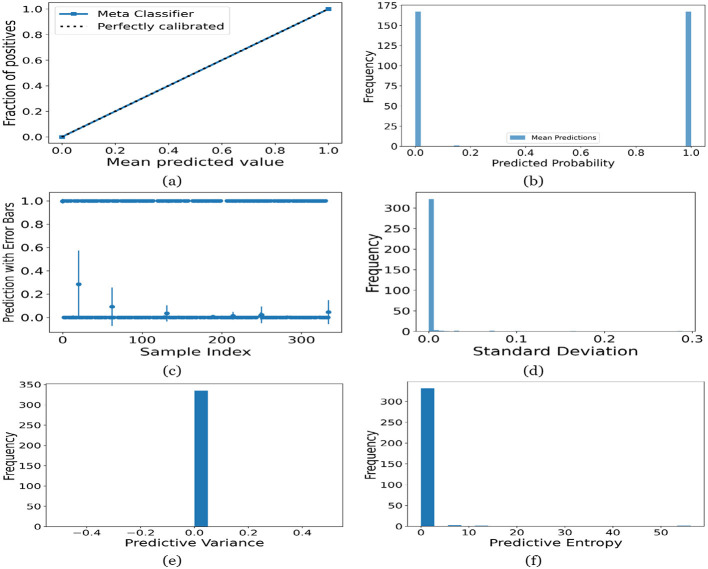
UA on Merged dataset utilizing **(a)** calibration curve, **(b)** mean probability bar graph, **(c)** predictive error bar graph, **(d)** predictive standard deviation, **(e)** predictive variance, and **(f)** predictive entropy.

Figures 12–16 present the UA generated using MCD on both meth classifiers for Toddler, Child, Adolescent, Adult and Merged dataset, respectively. Each figure is comprised of six subplots, including (a) the calibration curve, (b) the predicted class probability plot, (c) the scatter plot with an error bar, (d) the standard deviation distribution, (e) the predictive variance distribution, and (f) the predictive entropy distribution. A calibration curve is a graphical representation that compares the mean predicted value of a probabilistic classifier with the actual fraction of positives. This plot is typically used to assess the reliability of probabilistic predictions made by a model. A well-calibrated model means that if the model predicts an event with a probability of “p”, then “p” percent of the time that event should occur. If the model is perfectly calibrated, the plot of the model's predictions would lie on the diagonal line representing the “Perfectly calibrated” classifier. Deviations from this line indicate a model whose probabilities are either over- or under-confident.

For the Toddler dataset, the UA is presented in Figures 12. Firstly, the calibration curve is provided in Figure 12a. The reference line, labeled “Perfectly calibrated”, is a dotted line that forms a 45-degree angle, indicative of a hypothetical model where the predicted probabilities perfectly match the observed proportions. The “Meta Classifier” calibration curve closely follows the “Perfectly calibrated” reference line, suggesting that the classifier's predicted probabilities are well-calibrated. The squares on the “Meta Classifier” line potentially represent binned average predictions compared to the actual outcomes within those bins. Secondly, the predicted class probability is provided in Figure 12b. There are two prominent peaks, one at the extreme left (near 0.0) and one at the extreme right (near 1.0), which means the model is often very confident in its predictions, assigning probabilities close to 0 or 1. The frequency of predicted probabilities near 0.0 is slightly higher than those near 1.0. This suggests that the model predicts the negative class more frequently than the positive class or that there are more instances of the negative class in the dataset. A noticeable absence of predicted probabilities in the middle range (from 0.2 to 0.8) indicates that the model rarely assigns intermediate probabilities and is generally certain about its predictions. Figures 12c presents the scatter plot with error bars. The scatter plot illustrates the predictions and associated uncertainties for a series of samples. Predominantly, the predictions align with absolute certainty at 0.0 and 1.0, implying high confidence in these outcomes. A few predictions demonstrate considerable uncertainty, as evidenced by the longer error bars. These instances of increased uncertainty are interspersed without a clear pattern across the sample index. The visualization highlights the model's confidence in most predictions while acknowledging uncertainty in a subset of cases. This underscores the importance of accounting for error margins in predictive analysis, especially when utilizing these predictions for further decision-making processes. Lastly, Figures 12d–f present the distribution of predictive standard deviation, variance, and entropy. In Figure 12d, most of the data points have a very low standard deviation, close to 0.00. This is indicated by the tall bar at the extreme left of the histogram. There is a rapid decrease in frequency as the standard deviation increases. After the initial tall bar, subsequent bars are significantly shorter, showing that higher standard deviations are much less common in this dataset. The distribution is heavily skewed to the left, meaning that there is a higher concentration of lower standard deviation values and very few high standard deviation values. In Figure 12e, the histogram displays the distribution of predictive variances from a set of model predictions. A pronounced peak at a predictive variance of 0.0 indicates that nearly all predictions have no variance, implying a high degree of certainty or consistency in the model's output. The absence of visible frequencies for non-zero variances suggests either an absence or an insignificant number of predictions with any measurable uncertainty. In Figure 12f, there is a significant concentration of predictions with an entropy close to 0.0, as evidenced by the tall bar at the beginning of the histogram. This suggests that for many predictions, the model is very certain about the outcome. The frequency of predictions decreases sharply as entropy increases. There are very few predictions with higher entropy values, which would indicate uncertainty in the model's predictions. The histogram does not show any occurrences of predictions with entropy near 1.0. This would mean there are no instances where the model is completely uncertain about the outcome. Figures 13–16 can be interpreted in the similar way.

We assessed the impact of MCD, which quantifies prediction uncertainty. Removing MCD resulted in reduced reliability, as the model failed to estimate confidence in its predictions. This highlighted the importance of incorporating uncertainty analysis to make the framework robust and trustworthy for clinical applications.

## 7 Conclusion

In conclusion, this study proposed a novel ensemble learning framework for the classification of ASD using questionnaire data, integrating Safe-Level SMOTE for class imbalance handling, SHAP for model interpretability, and MCD for uncertainty estimation. Compared to baseline ML methods, the model demonstrated exceptional performance across five publicly available datasets representing multiple developmental stages, with high accuracy, transparency, and reliability.

However, this study has certain limitations that warrant further consideration. From a theoretical perspective, the reliance on questionnaire data introduces subjectivity and potential reporting biases, which may affect the accuracy and generalizability of the model. Additionally, while the stacked ensemble learning approach leverages the strengths of multiple classifiers, it requires significant computational resources for both training and hyperparameter tuning. This may limit its deployment in environments where computational power is constrained. Another theoretical limitation lies in the binary classification setup, as this study does not yet address the multi-classification of ASD severity, which could provide more granular insights into the disorder.

From a practical standpoint, the datasets utilized, while publicly accessible and diverse across age groups, may not comprehensively represent the full heterogeneity of the ASD population, particularly across different geographical and socio-demographic backgrounds. Furthermore, the lack of real-world clinical validation is a significant limitation, as the model's robustness and reliability have not yet been tested within practical healthcare workflows or clinical environments.

To address these limitations, future work will explore integrating hybrid metaheuristic optimization techniques, such as swarm intelligence, to enhance feature selection and model performance. Incorporating multi-modal data, including neuroimaging and behavioral assessments alongside questionnaire data, can further strengthen the predictive capability and generalizability of the framework. Additionally, collaboration with clinicians will enable real-world validation and facilitate the model's integration into clinical decision-making systems. Extending the current binary classification model to handle multi-class classification tasks will allow for more refined predictions, such as ASD severity levels or subtypes. Finally, efforts will be made to optimize the model for lightweight deployment, ensuring accessibility in real-time systems and resource-limited environments.

By addressing these theoretical and practical limitations through the outlined future directions, the proposed framework has the potential to evolve into a reliable, scalable, and clinically applicable tool for early ASD detection, contributing to improved diagnostic capabilities and better outcomes for individuals with ASD.

## Data Availability

Publicly available datasets were analyzed in this study. This data can be found here: https://archive.ics.uci.edu/dataset/426/autism+screening+adult and https://archive.ics.uci.edu/dataset/419/autistic+spectrum+disorder+screening+data+for+children.
