# Supplementary material for: Early diagnosis of autism across developmental stages through scalable and interpretable ensemble model
Source: Front Artif Intell. 2025 May 30;8:1507922. doi: 10.3389/frai.2025.1507922 (PMC12164165; doi:10.3389/frai.2025.1507922)
Supplement: Supplementary file 1 [file Data_Sheet_1.pdf]

# Supplementary Material

## 1 DATA PREPROCESSING

The initial stage in developing an ML model is data preparation, which marks the start of the process. Real-world data is frequently inadequate, inconsistent, imprecise (including errors or anomalies), and missing specific attribute values/trends. As a result, data preparation is a critical phase in the ML pipeline, as it involves cleaning, transforming, and normalizing data to make it suitable for analysis and training models. Figure S1 depicts the data preprocessing stages of this work. In this work, we have utilized various data preprocessing techniques, including:

1. Data Cleaning: We have begun by searching for any form of missing value in the datasets listed below. Missing values have been found in all the datasets' " age " columns. We have replaced the missing values using mean/median imputation technique Zhang (2016) Jadhav et al. (2019).
2. Categorical Data Encoding: Categorical data encoding converts categorical variables into numerical variables, which can be used in ML models. Categorical data consists of variables that are organized into distinct categories. Since most ML models are based on mathematical equations, categorical data must be converted to numerical data to avoid complications. So, we have encoded the values in "ASD Traits" column that contained categorical data (No, Yes) into numerical values(0,and 1). For this purpose, we have used the LabelEncoder() method from sklearn library Bisong and Bisong (2019).
3. Handling Class Imbalance: We have utilized Safe-level-SMOTE to address the class imbalance. Synthetic Minority Over-sampling Technique(SMOTE) is a method used to address class imbalance in datasets, particularly in the context of supervised learning Chawla et al. (2002). It works by creating synthetic samples from the minority class rather than copies, which helps overcome the overfitting problem of random oversampling. Traditional oversampling techniques, such as SMOTE, can introduce synthetic samples to balance class distribution but may inadvertently generate outliers or noisy instances, potentially deteriorating model performance. Safe-Level SMOTE, proposed by Bunkhumpornpat et al. Bunkhumpornpat et al. (2009), addresses this issue by prioritizing the generation of synthetic samples in regions of the feature space that are deemed "safe" based on their proximity to existing minority class instances. The key advantage of Safe-Level SMOTE lies in its ability to balance class distribution while mitigating the risk of introducing synthetic outliers. By focusing on regions of the feature space that are densely populated by minority class instances, Safe-Level SMOTE preserves the integrity of the minority class distribution and enhances the robustness of ML models trained on imbalanced biomedical datasets. Figure **??** presents the data distribution in the datasets after performing SMOTE and Safe-level-SMOTE.
4. Feature Importance Calculation: When constructing predictive models, it is critical to comprehend the significance of every feature in relation to the target variable. A productive approach to assess this level of importance is the computation of Mutual Information (MI) scores. In contrast to more straightforward linear metrics, MI offers a broader measure that can encompass any relationship between variables, whether it be nonlinear or linear Liu and Motani (2020). If two variables are independent, then the score is zero. Conversely, a more significant score signifies an enhanced interdependence or correlation among the variables. In the context of feature selection, the MI score of a feature concerning the target variable indicates the degree to which knowledge of the feature reduces uncertainty regarding the target. MI scores were utilized to ascertain the features within the dataset

that possess the most significant relevance to the objective variable. This method proved particularly advantageous in our particular scenario, where our dataset comprised a combination of linear and non-linear relationships that the MI technique could accurately capture. The ranking of the features according to their MI scores provided a distinct perspective on which features might serve as more significant predictors of the target variable. The model could be simplified by identifying and retaining solely the most informative features, thereby mitigating the potential for overfitting and enhancing interpretability. Figure S2, S3, S4, S5, S6 provides the feature importance bar graph generated using MI score for Toddler, Child, Adolescent, Adult, and Merged dataset respectively.

5. Feature Correlation Analysis: A critical aspect of statistical analysis and data exploration is the comprehension of the interrelationships among variables. In pursuit of this objective, we utilized the Pearson correlation coefficient, a commonly employed technique for quantifying the linear association between two continuous variables within our dataset. The range of values for the Pearson correlation coefficient (r) is -1 to +1. The coefficient's magnitude denotes the correlation's intensity, whereas the sign indicates the direction. The Pearson correlation method was utilized to examine the relationships between every pair of continuous variables in the dataset Obilor and Amadi (2018) Sedgwick (2012). By identifying substantially correlated variables, one can acquire valuable insights into latent patterns and connections that may indicate the dataset's intrinsic causal relationships or interdependencies. A strong correlation between two variables could potentially signify duplication of effort. When this occurs, one of the variables can be omitted to decrease dimensionality without significant information loss, thus streamlining our model without compromising its ability to make predictions. The results obtained from the Pearson correlation analysis contributed substantially to our comprehension of the dataset, enabling us to make more informed decisions during the subsequent modeling phases. Figure S7, figure S8, figure S9, figure S10, and figure S11 presents the feature correlation heatmap for Toddler, Child, Adolescent, Adult, and Merged dataset respectively.
6. Feature Reduction: The feature selection process is crucial in determining the performance and interpretability of a model. We utilized Principal Component Analysis (PCA) for feature reduction technique. PCA is a widely used dimensionality reduction technique that aims to transform high-dimensional data into a lower-dimensional space while preserving as much of the original variance as possible. PCA has become a fundamental tool in various fields, including biomedical data analysis, where datasets often exhibit high dimensionality due to numerous features or variables. PCA can reduce the dimensionality of high-dimensional biomedical datasets while retaining important information, facilitating visualization, interpretation, and computational efficiency. PCA can aid in identifying the most informative features or variables in the dataset by examining the contributions of each feature to the principal components. PCA can help mitigate the effects of noise and redundancy in biomedical data, leading to improved signal-to-noise ratios and enhanced interpretability Jolliffe and Cadima (2016). It can be difficult to ascertain the optimal number of features (k). Thus, we employed feature correlation and feature importance techniques. Finally, we selected 12 as the optimal number of features.

## 2 SUPPLEMENTARY TABLES AND FIGURES

## REFERENCES

Bisong, E. and Bisong, E. (2019). Introduction to scikit-learn. *Building Machine Learning and Deep Learning Models on Google Cloud Platform: A Comprehensive Guide for Beginners* , 215–229

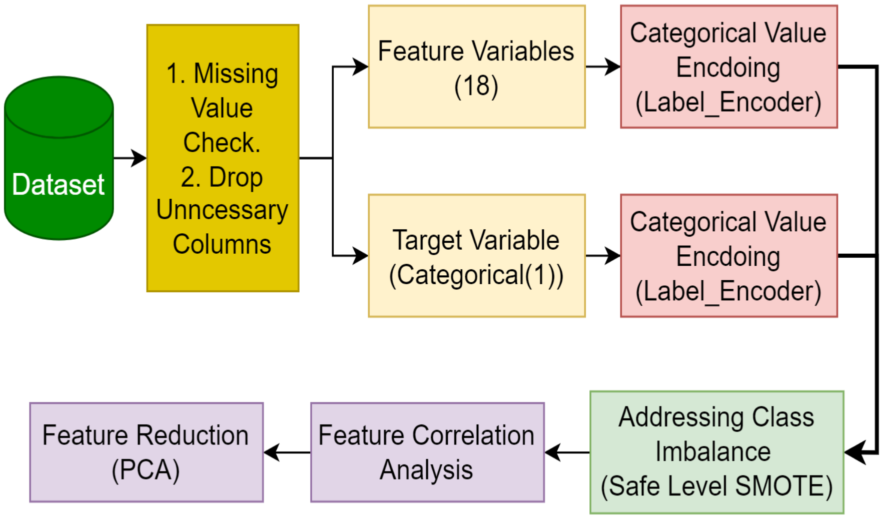


**Figure S1.** The stages of data preprocessing.

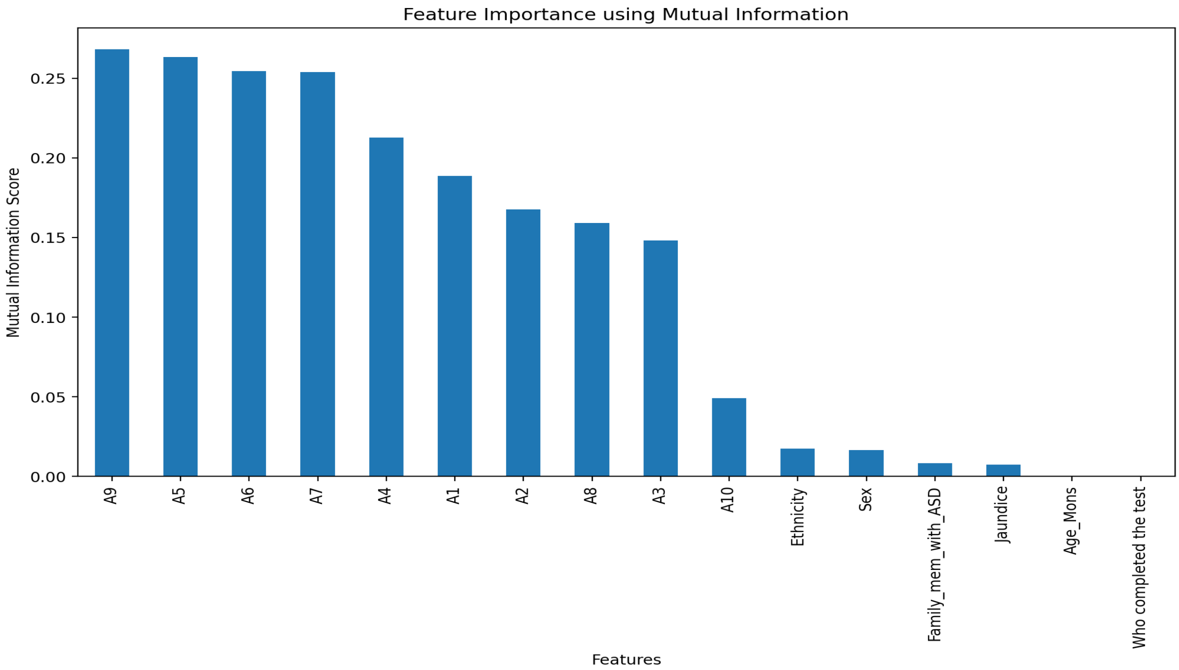


**Figure S2.** Feature importance bar graph of Toddler dataset.

Bunkhumpornpat, C., Sinapiromsaran, K., and Lursinsap, C. (2009). Safe-level-smote: Safe-level-synthetic minority over-sampling technique for handling the class imbalanced problem. In *Advances in Knowledge Discovery and Data Mining: 13th Pacific-Asia Conference, PAKDD 2009 Bangkok, Thailand, April 27-30, 2009 Proceedings 13* (Springer), 475–482

Chawla, N. V., Bowyer, K. W., Hall, L. O., and Kegelmeyer, W. P. (2002). Smote: synthetic minority over-sampling technique. *Journal of artificial intelligence research* 16, 321–357

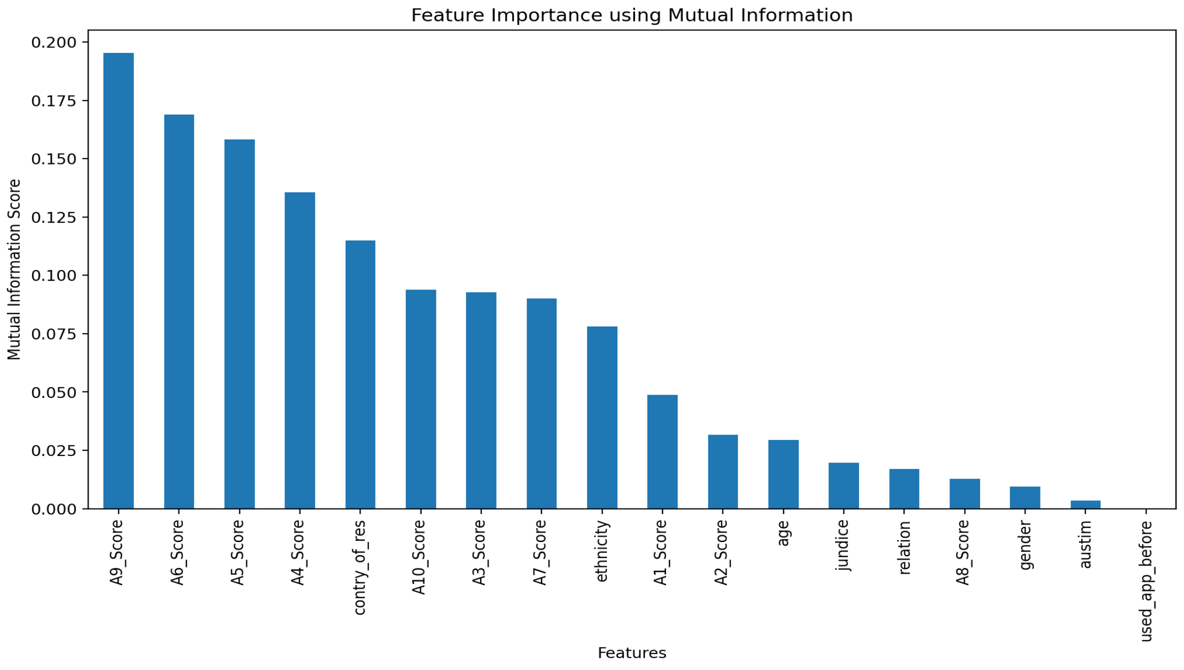


**Figure S3.** Feature importance bar graph of Child dataset.

Jadhav, A., Pramod, D., and Ramanathan, K. (2019). Comparison of performance of data imputation methods for numeric dataset. *Applied Artificial Intelligence* 33, 913–933

Jolliffe, I. T. and Cadima, J. (2016). Principal component analysis: a review and recent developments. *Philosophical transactions of the royal society A: Mathematical, Physical and Engineering Sciences* 374, 20150202

Liu, S. and Motani, M. (2020). Exploring unique relevance for mutual information based feature selection. In *2020 IEEE International Symposium on Information Theory (ISIT)* (IEEE), 2747–2752

Obilor, E. I. and Amadi, E. C. (2018). Test for significance of pearson's correlation coefficient. *International Journal of Innovative Mathematics, Statistics & Energy Policies* 6, 11–23

Sedgwick, P. (2012). Pearson's correlation coefficient. *Bmj* 345

Zhang, Z. (2016). Missing data imputation: focusing on single imputation. *Annals of translational medicine* 4

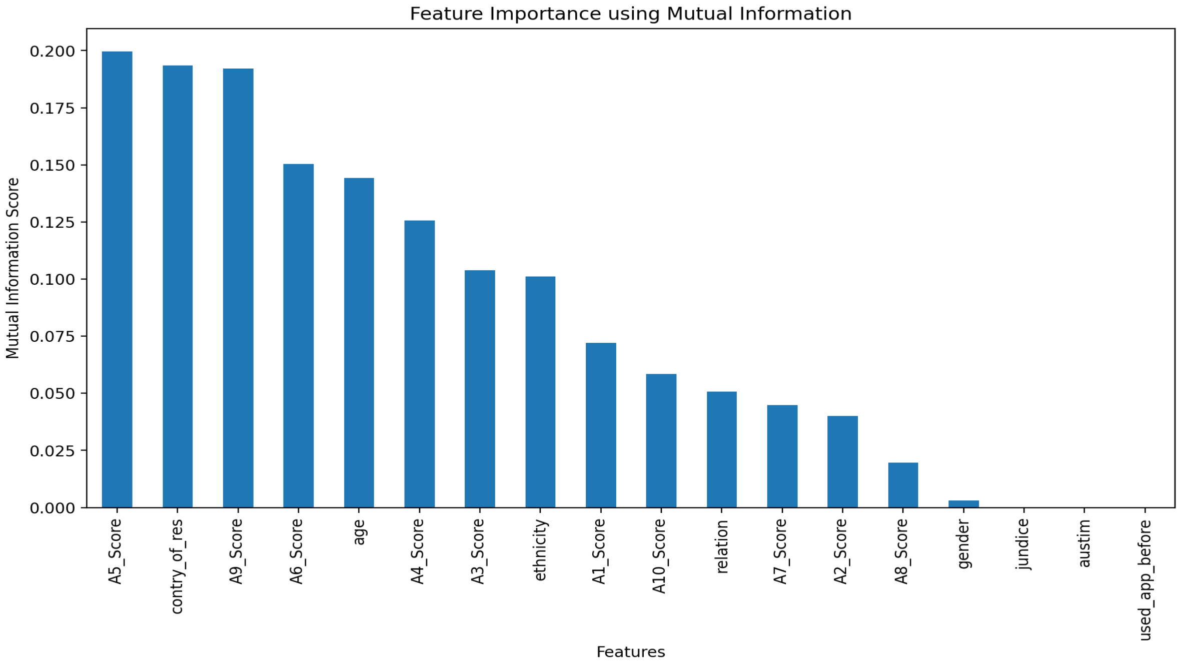


**Figure S4.** Feature importance bar graph of Adolescent dataset.

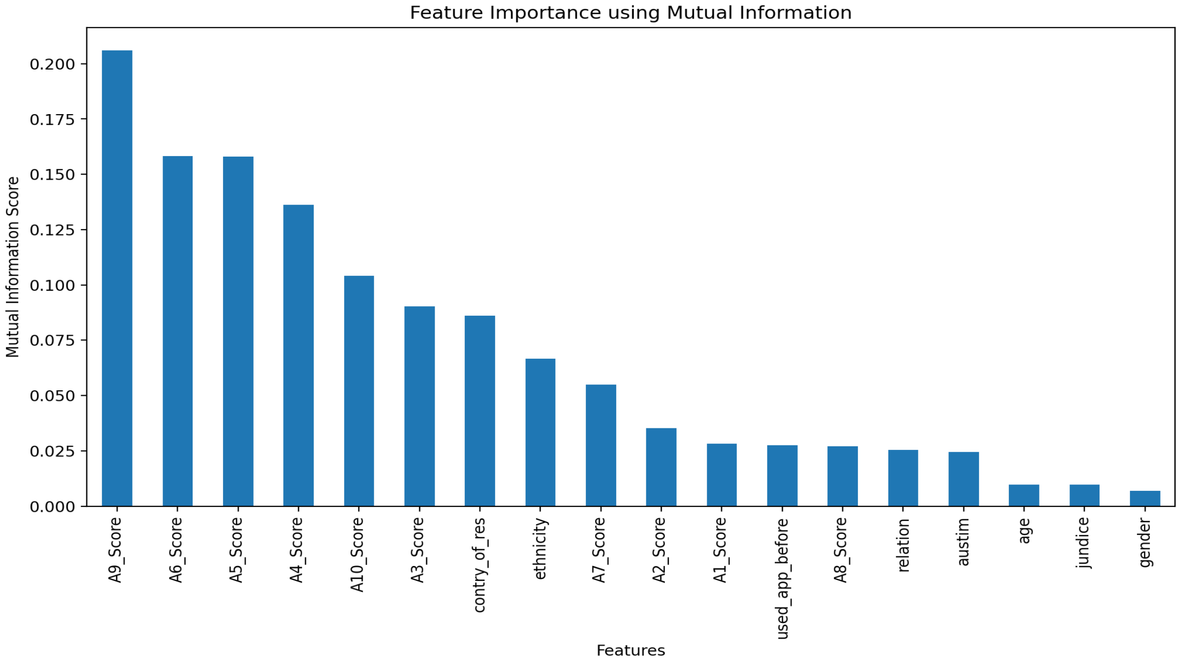


**Figure S5.** Feature importance bar graph of Adult dataset.

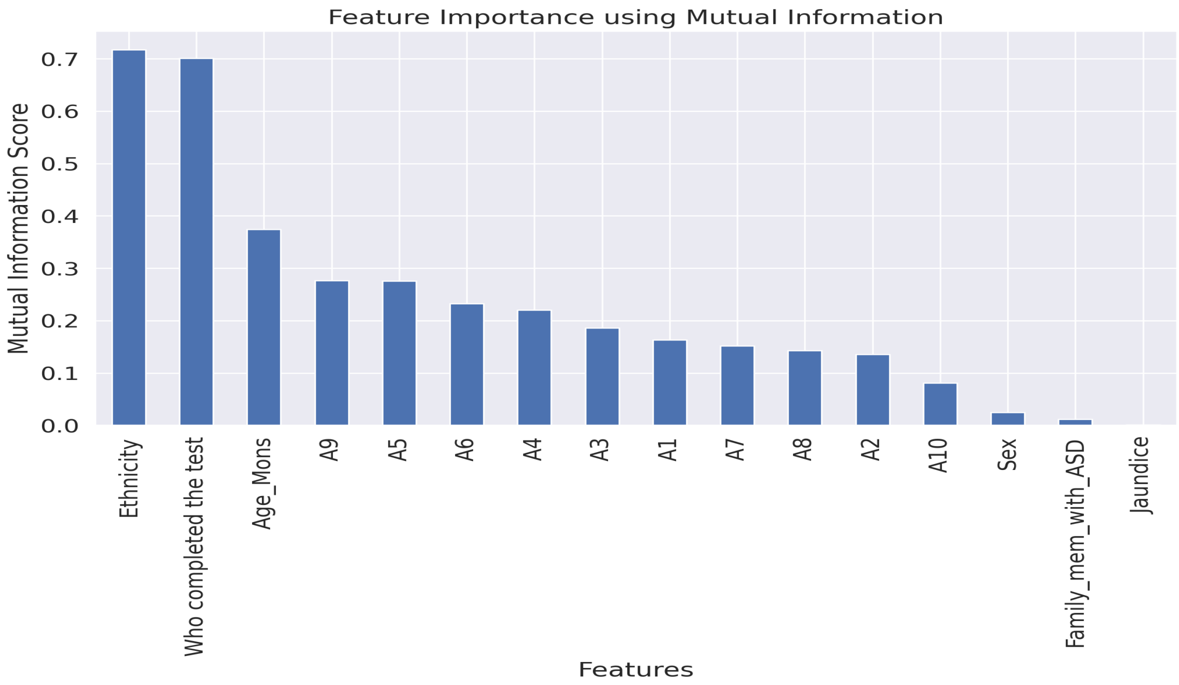


**Figure S6.** Feature importance bar graph of Merged dataset.

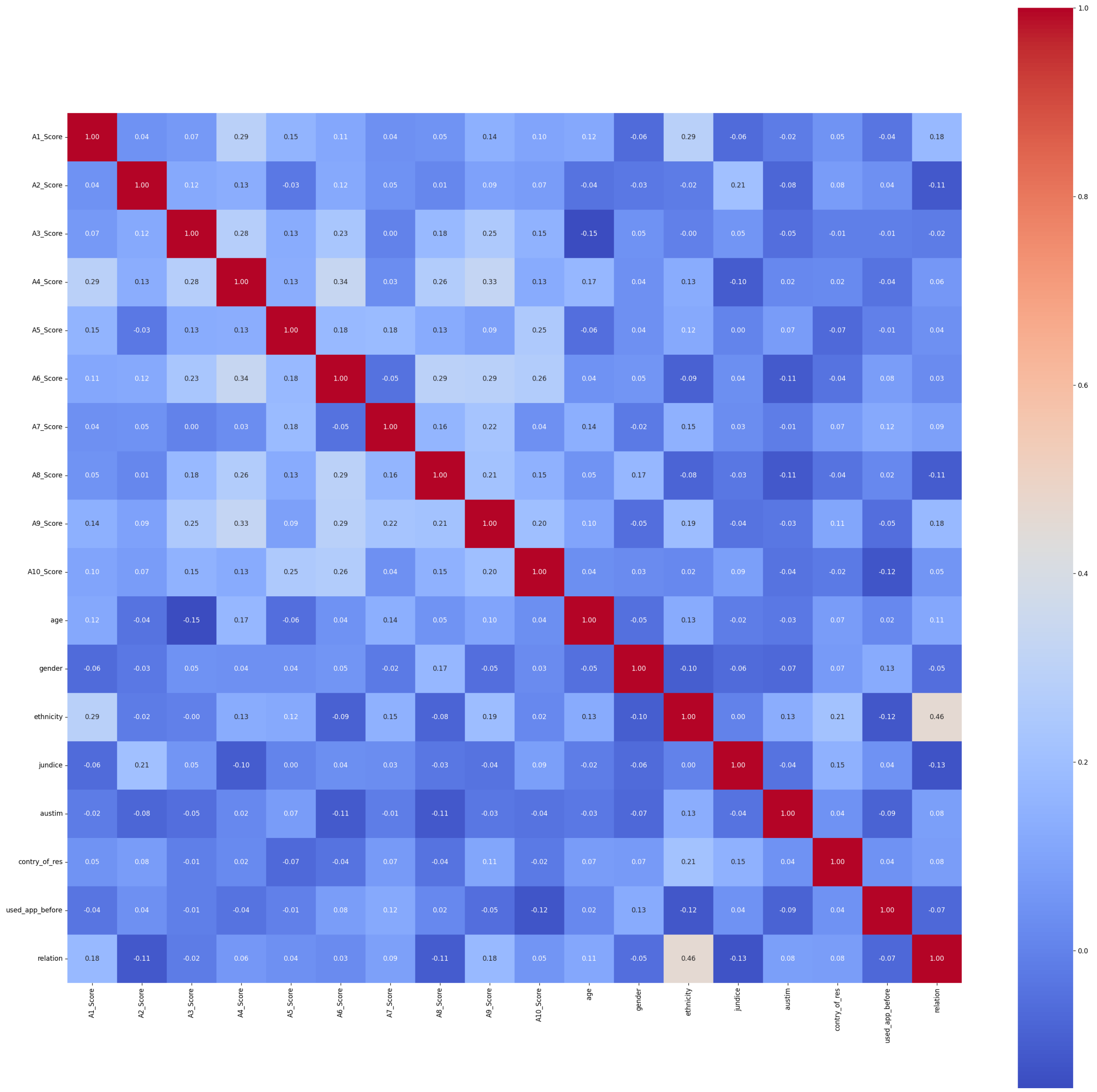


**Figure S7.** Feature correlation heatmap of Toddler dataset.

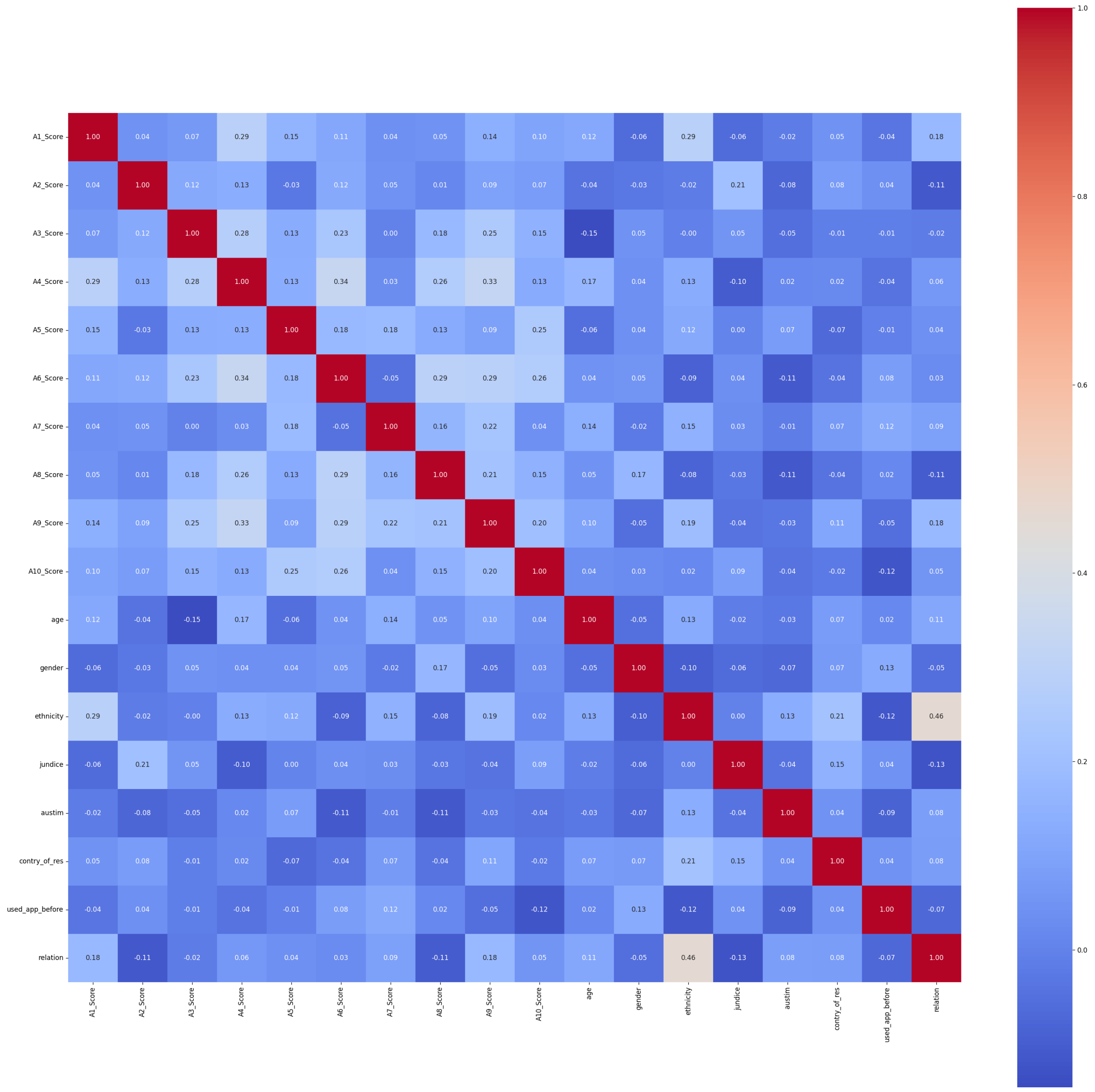

**Figure S8.** Feature correlation heatmap of Child dataset.

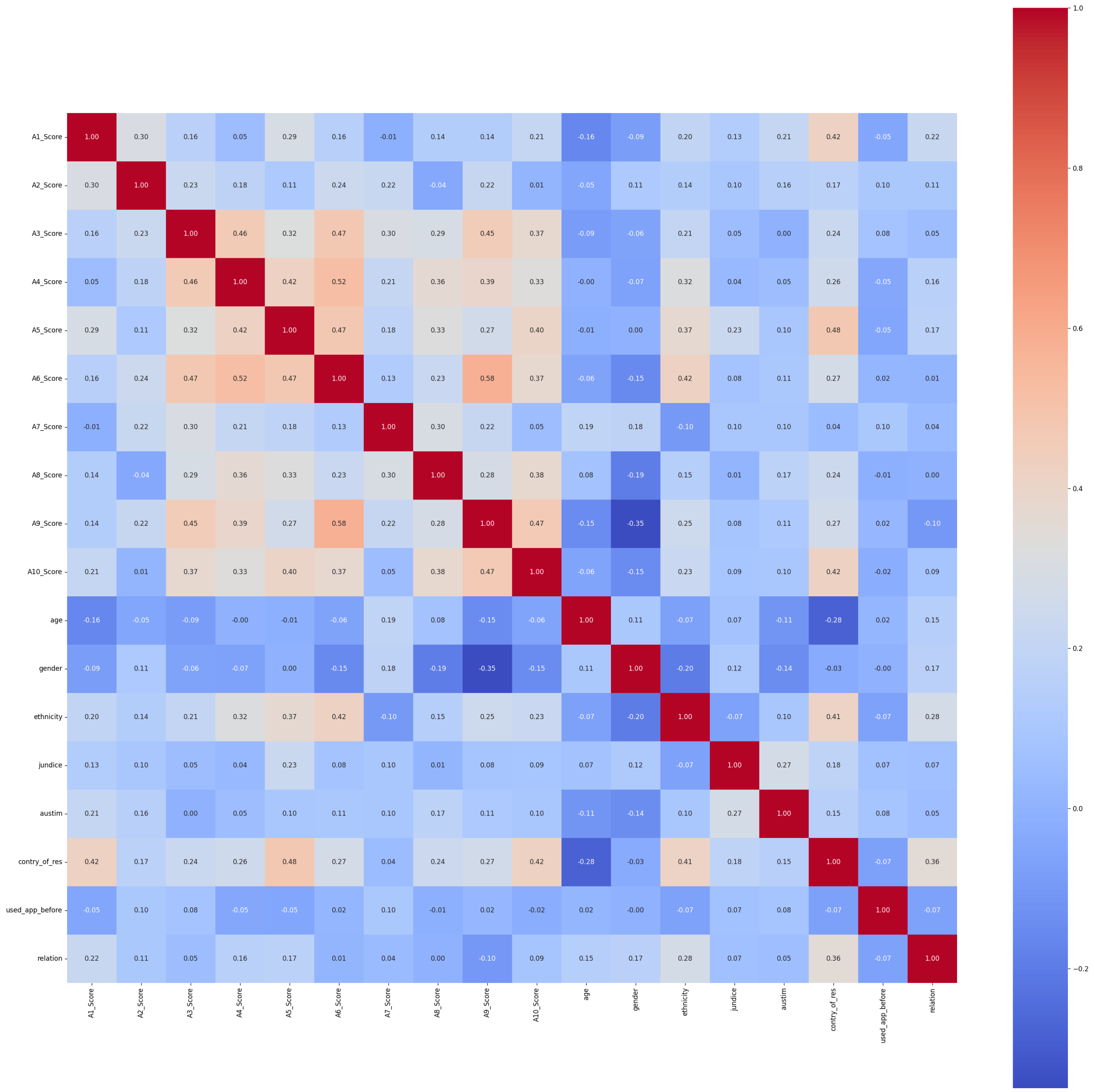


**Figure S9.** Feature correlation heatmap of Adolescent dataset.

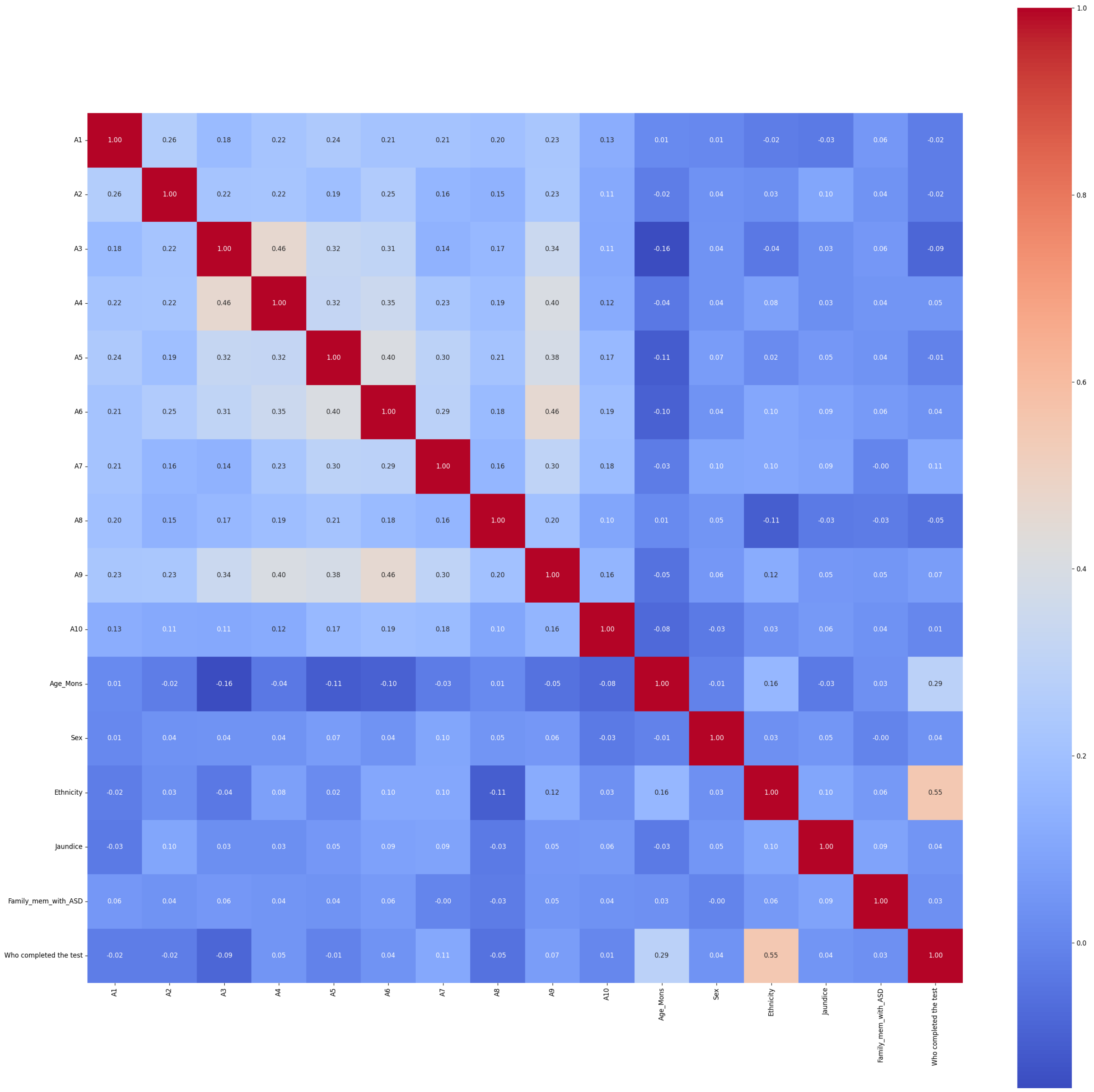


**Figure S10.** Feature correlation heatmap of Adult dataset.

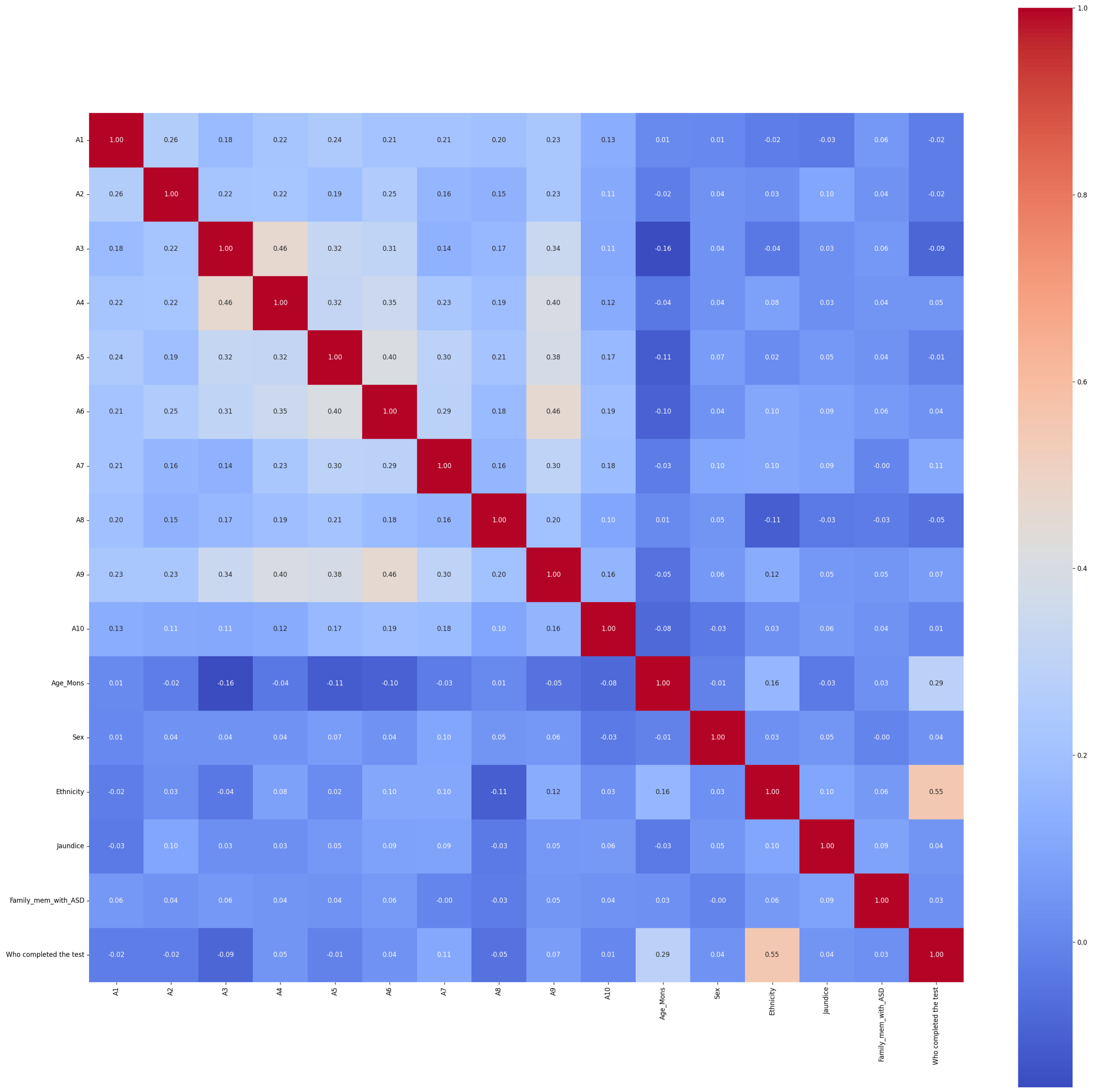


**Figure S11.** Feature correlation heatmap of Merged dataset.
